# NAD^+^ Metabolism and Immune Regulation: New Approaches to Inflammatory Bowel Disease Therapies

**DOI:** 10.3390/antiox12061230

**Published:** 2023-06-07

**Authors:** Chaoyue Chen, Wei Yan, Meihui Tao, Yu Fu

**Affiliations:** 1 Department of Gastroenterology, Union Hospital, Tongji Medical College, Huazhong University of Science and Technology, Wuhan 430022, China; 2 Department of Gastroenterology, Tongji Hospital, Tongji Medical College, Huazhong University of Science and Technology, Wuhan 430022, China

**Keywords:** NAD^+^, inflammatory bowel disease, immune regulation, mitochondria, intestinal epithelial barrier, intestinal stem cells

## Abstract

Inflammatory bowel disease (IBD), which includes Crohn’s disease (CD) and ulcerative colitis (UC), is a multifactorial systemic inflammatory immune response. Nicotinamide adenine dinucleotide (NAD^+^) is a co-enzyme involved in cell signaling and energy metabolism. Calcium homeostasis, gene transcription, DNA repair, and cell communication involve NAD^+^ and its degradation products. There is a growing recognition of the intricate relationship between inflammatory diseases and NAD^+^ metabolism. In the case of IBD, the maintenance of intestinal homeostasis relies on a delicate balance between NAD^+^ biosynthesis and consumption. Consequently, therapeutics designed to target the NAD^+^ pathway are promising for the management of IBD. This review discusses the metabolic and immunoregulatory processes of NAD^+^ in IBD to examine the molecular biology and pathophysiology of the immune regulation of IBD and to provide evidence and theoretical support for the clinical use of NAD^+^ in IBD.

## 1. Introduction

Inflammatory bowel disease (IBD) is a complex systemic inflammatory immune response caused by multiple factors. Crohn’s disease (CD) and ulcerative colitis (UC) are two types of IBD. IBD continues to strain medical resources and increase the risk of colorectal cancer worldwide [1,2]. Understanding the pathophysiology, molecular, and biochemical mechanisms of IBD could help in its early detection and management. Recent studies have provided compelling evidence regarding the significant role of immune metabolism in the development of immunological disorders, notably inflammatory bowel disease (IBD). The complex interplay between metabolism and the pathogenesis of IBD highlights the potential of manipulating metabolic signals to modulate immune processes [3]. Consequently, there has been a surge of interest in exploring personalized targeted biological therapies for IBD, benefiting from remarkable advancements in metabolic research and the availability of sophisticated tools such as metabolomics. These tools have contributed to a deeper understanding of the intricate network of mitochondrial metabolism and bioenergy, which are crucial regulatory factors for maintaining intestinal homeostasis [4,5]. These noteworthy breakthroughs hold great promise for the development of innovative approaches for IBD treatment, specifically by targeting immune metabolism.

Nicotinamide adenine dinucleotide (NAD^+^) is found in all living cells and plays many roles in cell integrity [6,7]. In addition to energy metabolism, NAD^+^ and its breakdown products affect calcium homeostasis, gene transcription, DNA repair, and cell-to-cell communication [8]. NAD^+^ and the reduced form of nicotinamide adenine dinucleotide (NADH) are the key metabolic regulators supplying the mitochondrial respiratory chain with protons and electrons to form adenosine triphosphate (ATP) [9]. NAD^+^-dependent signaling regulates gene expression and metabolic activity by mediating changes in energy homeostasis [10].

Despite the increase in research interest in immunometabolism over the past decade, the specific effects of NAD^+^ on immune cell function and inflammatory responses have yet to be fully elucidated. NAD^+^ metabolism determines the development of inflammatory diseases and has been widely explored for its therapeutic and preventive potential in various preclinical models and diseases. The NAD^+^ precursor NR is bioavailable to the human skeletal muscle as a nutritional supplement and exhibits exceptional immunomodulatory properties [11]. Increasing NAD^+^ levels prevent cardiomyopathy by inhibiting the pro-inflammatory activation of circulating immune cells, thereby improving clinical outcomes [12]. Increasing NAD^+^ and GSH levels may serve as viable treatments for coronavirus [13,14]. The immunomodulatory capacity of NR can assist in alleviating disease progression and reducing disease severity in COVID-19 patients with pneumonia [15]. NAD^+^ inhibits or enhances the immune response depending on the functional differences in NAD^+^-dependent enzymes. By controlling the flexibility of immune regulation through NAD^+^, an essential new model can emerge as an effective therapeutic application of NAD^+^ biology and a better understanding of the role of NAD^+^ homeostasis in various types of inflammatory diseases.

**Figure 1:**
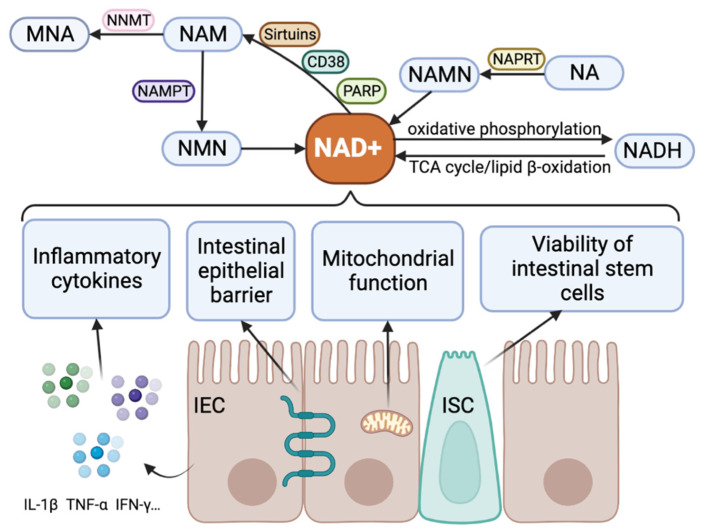
Metabolic processes of NAD^+^ and the biological function of NAD^+^ in IBD. The metabolism of NAD^+^ is affected by enzymes such as sirtuins, CD38, PARP, NAMPT, NAPRT, NNMT, etc. The interconversion of NAD^+^ and NADH is achieved through oxidative phosphorylation, TCA cycling, and lipid β-oxidation. NAD^+^ metabolism regulates the immune processes of IBD through inflammatory cytokines, intestinal epithelial barrier, mitochondrial function, the viability of intestinal stem cells, etc. Abbreviations: CD38, cluster of differentiation 38; IEC, intestinal epithelial cell; IFN-γ, interferon gamma; IL-1β, interleukin 1 beta; ISC, intestinal stem cell; MNA, methylnicotinamide; NA, niacin; NAD^+^, nicotinamide adenine dinucleotide; NADH, reduced form of nicotinamide adenine dinucleotide; NAM, nicotinamide; NAMN, nicotinic acid mononucleotide; NAMPT, nicotinamide phosphoribosyl transferase; NAPRT, nicotinate phosphoribosyl transferase; NMN, nicotinamide mononucleotide; NNMT, nicotinamide N-methyltransferase; PARP, poly ADP-ribose polymerase; TCA, tricarboxylic acid; TNF-α, tumor necrosis factor alpha.

Many studies have linked NAD^+^ metabolism to inflammatory diseases, intestinal homeostasis, and IBD. The acute inflammatory state of the body has a direct impact on serum NAD^+^ levels, subsequently influencing the systemic inflammatory phenotype [16]. Notably, “niacin and niacinamide metabolism” emerges as the predominant metabolic characteristic of inflamed tissues in ulcerative colitis (UC) [17]. Intestinal homeostasis of IBD requires a balance between NAD^+^ production and depletion [18]. The heightened activity of NAD^+^-consuming enzymes in IBD has been implicated in the onset of intestinal inflammation [18]. Consequently, elevating NAD^+^ levels or activating sirtuins may have protective effects on the gut barrier, potentially preventing the initiation or progression of IBD. Pharmacological precursors of NAD^+^ have shown promise in regulating inflammation and hold potential as therapeutic interventions for inflammatory diseases [19]. Therefore, the development of drugs targeting the NAD^+^ pathway holds promise for controlling IBD. Here, we summarized metabolic processes of NAD^+^ and the biological function of NAD^+^ in IBD (Figure 1).

## 2. Immunometabolism and Inflammatory Bowel Disease

Previous studies have underscored the significance of immune cell subsets in the context of IBD. Notably, disparities in mucosal T cell subsets among IBD patients have demonstrated a potential diagnostic value in predicting responses to distinct therapeutic interventions. Moreover, specific intestinal mucosal T lymphocyte subsets hold promise as potential biomarkers during the course of IBD, offering valuable insights into disease progression and treatment outcomes [20]. The study of immune metabolism is a relatively new scientific field in IBD. Immune metabolism regulates the activation, proliferation, and acquisition of effector functions and homeostasis by modulating gene expression, epigenetic remodeling, and post-translational modifications [21]. Several studies have reported that the environment, including growth factors, nutrient availability, and intracellular processes, including internal metabolites, reactive oxygen species (ROS), and reduction/oxidation substrates influence immune cell metabolic pathways [22,23,24]. Recent studies have shown that immune metabolism is the basis of life and the root cause of many immune-related diseases, including IBD. Since metabolism shapes the pathogenesis of IBD, manipulating metabolic signals can be used to control the immune process [3]. The reversible plasticity of effector cells within the innate and adaptive immune systems opens avenues for the development of novel therapies for inflammatory diseases. Epigenetic reprogramming plays a pivotal role in regulating immunity, metabolism, and mitochondrial bioenergetics, thereby exerting control over inflammatory processes [19]. Metabolic pharmacology may treat inflammatory conditions [25,26].

Dysfunction of the epithelial barrier and dysregulation of the mucosal immune response characterize IBD, which includes UC and CD [27]. It is a complex, chronic, recurrent inflammatory immune response of the gastrointestinal tract caused by various factors [28]. IBD is caused by altered intestinal immune responses that are influenced by host microbiota and genetic susceptibility [4,5]. Long-term, self-perpetuating intestinal inflammation may result in colorectal cancer (CRC) [29].

New personalized targeted biological therapies for IBD have been investigated in recent years. However, many patients remain unresponsive, and adverse drug reactions or loss of secondary drug reactions have resulted in the need for surgery in the later stages of disease development. Several immunotherapy checkpoint inhibitors used for cancer treatment are associated with several adverse effects on pre-existing IBD in patients [30]. Mitochondrial metabolism and bioenergy have emerged as key regulators of host intestinal homeostasis due to recent advances in metabolic research and the development of tools such as metabolomics [31,32]. Immune metabolism is a new potential method for treating IBD. Immune cells in IBD have distinct metabolic properties at different stages of the immune response, affecting their ability to proliferate, differentiate, and function [3]. Immune metabolism regulates the immune response, which could partly explain the pathogenesis of IBD and provide a theoretical foundation for immunometabolic therapy and new approaches to treating IBD.

## 3. NAD^+^ Metabolism

British biochemists Arthur Harden and William John Young discovered NAD^+^, the first cofactor, in 1906 [33]. Studies have found that NAD^+^ is present in all cells and is essential for many biological reactions [6,7]. Serine, adenosine diphosphate ribosyltransferase, and synthetase use it as a co-substrate and an important co-enzyme in cell signaling and energy metabolism [19].

Glycolysis, β oxidation, the tricarboxylic acid (TCA) cycle, and oxidative phosphorylation all involve redox reactions to NAD^+^/NADH [34]. It has been experimentally demonstrated that NADK adds phosphate to the adenosine ribose of NAD^+^ to produce NADP^+^ [8]. On the other hand, NADP^+^ and NADPH protect cells from oxidative stress and synthesize fatty acids, cholesterol, and DNA [8].

NAD^+^ is an important metabolic intermediate, a substrate for more than 300 enzymes, and a key regulator of many signaling pathways [35]. Enzymes interacting with NAD^+^ alter their activity, produce cell signaling molecules, or alter histones that suppress or initiate post-translational modifications of transcriptional proteins [7]. Studies have reported that NAD^+^ links cellular energy metabolism to downstream signaling and helps cells adapt to bioenergetic stress [36]. Tissue homeostasis requires adequate levels of NAD^+^ [37].

The synthesis of NAD^+^ involves five major precursors and intermediates: 1-tryptophan, 2-nicotinamide (NAM), 3-niacin (NA), nicotinamide nucleoside (NR), and 5-nicotinamide mononucleotide (NMN) [38], and can be produced from NAM, NR (remediation pathway), tryptophan (denovo pathway), and NA (Preiss–Handler pathway) [39]. They regulate most cellular activities. NAD^+^ can be broken down into NAM and adenosine diphosphate-ribose (ADPr) or its variants (2/3’-O-acyl-ADPr, polyADPr, or cyclic-ADPr) depending on the type of enzyme, including sirtuins, poly ADP-ribose polymerase (PARP), CD38, or CD157 [6]. Although nicotinamide phosphoribosyltransferase (NAMPT) and nicotinamide N-methyltransferase (NNMT) do not directly degrade NAD^+^, as enzymes control the NAD^+^ pathway, they catalyze NAD^+^ turnover, indirectly alter NAD^+^ concentrations, and regulate NAD^+^-related biological processes. It has also been observed that NAMPT converts NAM to the precursor of NAD^+^, NMN, counteracting the enzymes that break down NAD^+^, ensuring NAD^+^ availability [40]. On the other hand, NNMT methylates and excretes NAM, thereby lowering NAD^+^ precursors [41]. The activities of these enzymes are intricately regulated by the available levels of NAD^+^. As a result, NAD^+^-dependent signaling plays a crucial role in governing gene expression and metabolic activity, orchestrating changes in energy homeostasis [10].

Energy metabolism, DNA repair, regulation of the epigenetic landscape, and inflammation are all regulated by NAD^+^ metabolism [36,39,42]. Neurodegeneration [43], diabetes mellitus [44,45], obesity [46,47,48,49], heart disease [50,51], muscular dystrophy [52], renal dysfunction [53], and various types of cancers [39,54,55] are linked to changes in NAD^+^ metabolism. In addition, NAD^+^ is essential for energy homeostasis, metabolism, and signaling [19]. NAD^+^ pharmacological precursors can immunomodulate inflammation and enable therapeutic interventions in inflammatory diseases [19].

## 4. The Role of NAD^+^ in Regulating IBD

### 4.1. NAD^+^ and IBD

NAD^+^ metabolism and inflammatory diseases are being increasingly linked. Serum NAD^+^ levels were significantly elevated during inflammation. Murine serum NAD^+^ ranges from 0.1 to 0.5 micromoles physiologically, but during inflammation, levels of NAD^+^ in mice could increase up to 10 micromoles [16]. NAD^+^ has been implicated in the modulation of acute systemic inflammation, as it exerts regulatory control over immune and metabolic pathways in the context of sepsis [56]. The NAD^+^ salvage pathway is crucial for the inflammatory response to mount an appropriate response in LPS-induced monocytes [57]. In addition, researchers have found that NAD^+^ may maintain inflammatory states, activated immune systems, and cytokine storms by controlling NF-κB transcriptional activity [38,58,59].

NAD^+^ metabolism maintains intestinal homeostasis. Serum NAD^+^ levels increased three-fold in IBD patients compared to healthy people [18]. Metabolomic analysis of UC patients showed that “nicotinate and nicotinamide metabolism” was the most significant metabolic feature of UC-inflamed tissues, with a decrease in NAD^+^ levels and elevated levels of its metabolites NAM and ADPr. This suggests that NAD^+^ depletion in UC may result from increased NAD^+^ catabolism [17]. Although NAMPT is an enzyme in the NAD^+^ salvage pathway, proteomic profiles of proteins involved in NAD^+^ metabolism in IBD patients show that it is pro-inflammatory and tumorigenic [59].

IBD requires a balance between NAD^+^ biosynthesis and consumption to maintain intestinal homeostasis [18]. However, its pathogenesis is unknown [6]. Elevated activity of NAD^+^-consuming enzymes in IBD can cause gut inflammation [18]. In vitro and in vivo experiments have shown that NAD^+^ administration improves inflammation-related intestinal permeability by inhibiting NF-κB [60]. The gut microbiota provides alternative NAD^+^ synthesis pathways and enhances NAM or NR supplementation [61]. Therefore, biomedicine could utilize the gut microbiota to treat IBD by modulating NAD^+^ metabolism during intestinal inflammation. It was observed that NMN and NAD^+^ supplementation improved intestinal stem cell function in aged mice via mTOR and SIRT1 [62,63], and NMN prevented intestinal organoids from aging in old mice [64]. These results suggest that methods that increase NAD^+^ levels or activate sirtuins could protect the gut barrier and prevent IBD from starting or worsening. Nevertheless, it is important to note that augmenting NAD^+^ levels alone is not sufficient for preserving intestinal homeostasis entirely. Experimental evidence has shown that reducing NAD^+^ and SIRT1 levels in the colon of mice, using the olefin receptor agonist norisopodine (which expands epigenetic Treg cells as an aryl hydrocarbon receptor agonist), alleviates DSS-induced colitis [65]. As a result, drugs targeting the NAD^+^ pathway may help manage IBD.

### 4.2. NAD^+^ Metabolic Enzyme

The NAD^+^ metabolizing enzymes sirtuins, CD38, PARPs, NNMT, and NAMPT are linked to the inflammatory processes in IBD (Table 1).

#### 4.2.1. Sirtuins

Sirtuins are cofactors for the ADP-ribosylation reaction of NAD^+^ as deacetylators and substrates for NAD^+^-dependent enzymes [66]. In mammals, there are seven sirtuins (SIRT1-7) located at different subcellular locations, such as the cell nucleus (SIRT1, SIRT6, and SIRT7), cytoplasm (SIRT2), and mitochondria (SIRT3, SIRT4, and SIRT5) [136].

Of all the members in the sirtuins protein family, SIRT1 has received the most attention. Studies have reported that SIRT1 is an NAD^+^-dependent protein deacetylase that removes acetyl groups from lysine residues of the substrate protein [137]. In humans, SIRT1 has 741 amino acids, and despite the lack of a DNA-binding domain, it recruits the transcriptional machinery to target promoters and induces transcriptional changes [137]. Histones are major substrates of SIRT1, specifically H4K16Ac, and H3K9Ac [138,139]. The transcriptional regulation of SIRT1 depends on its gene and intracellular energy status [67,140,141]. Available evidence shows that SIRT1 deacetylates histones and non-histone substrates and regulates the circadian clock proteins [73,74,77], p53 [69], PPAR-γ [79], PGC1α [79], FOXO [142], and NF-κB [143]. The deacetylation of SIRT1, in turn, regulates the biological processes related to axonal integrity [67], such as cell differentiation [67], apoptosis [68], autophagy [70], development [71], and metabolism [72].

Extensive research has shown that sirtuins play distinct roles in chronic and acute inflammation. Chronic inflammatory diseases often consist of low sirtuin levels, whereas NAD^+^ and SIRT levels continue to decline in specific tissues, such as fat deposition in obesity and inflammation, brain in Alzheimer’s disease, and inflamed arteries in atherosclerosis [82,83]. Chronic inflammation decreases nuclear SIRT1 and SIRT6 levels/activity, increasing pro-inflammatory NF-κB RelA/p65 activity and gene expression [144]. Further research into the role of SIRT1 in chronic inflammation revealed that it increases NAD^+^ levels and rebalances metabolism and bioenergetics, restoring energy homeostasis in cells [145,146]. Compelling evidence is emerging to support the pivotal role of sirtuins as crucial regulators of immune cell inflammatory stress responses [147,148,149]. Sirtuins exert their influence through epigenetic modulation via histone and/or non-histone substrate deacetylation, consequently orchestrating the reprogramming of immune cell metabolism, phenotype, and bioenergetics [147,148,150,151]. The sirtuins/NAD axis serves as a critical interconnection point between epigenetics and metabolism, underscoring its significance as a fertile area of research with potential therapeutic implications [152].

Recent research has shown that SIRT1 reduces inflammation. First, it promotes the transition from glycolysis to fatty acid oxidation [153], suppresses the expression of PPAR-γ [154], and activates the key mitochondrial biogenesis transcription factor PGC-1β, thereby increasing oxidative metabolism [155]. The NLRP3 inflammasome activates Caspase-1 to cleave SIRT1 [156]. It has been experimentally shown that SIRT1 inhibits NLRP3 activation and increases NAD^+^ levels to reduce inflammation by preventing mitochondrial damage- induced ROS [80]. In addition, SIRT1 deacetylates and inactivates the NF-κB p65 subunit, restricting the expression of NF-κB-dependent pro-inflammatory genes [78]. SIRT1 knockdown experiments demonstrated the anti-inflammatory role of SIRT1 in macrophages and increased pro-inflammatory cytokines, specifically IL-1β [92]. Activation of TLR4 upregulates NAMPT, produces NAD^+^, and activates SIRT1 [81]. The nuclear-mitochondrial triad induces SIRT6 and SIRT3 in response to SIRT1 nuclear activation, switching from glycolysis to fatty acid oxidation and immunity from activation to inhibition during acute inflammation [84]. SIRT1 relies on the control of metabolic reprogramming of the immune system during adaptation. The available data show that under severe stress in sepsis, sustained NAD^+^ production and increased SIRT1 expression and activation maintain an adaptive phenotype. Blocking SIRT1 in sepsis mice during adaptation restores immunity, rebalances mitochondrial bioenergy, and improves survival [84]. Thus, these findings imply a strong link between SIRT1 activity regulation and inflammation.

There are conflicting conclusions in the literature regarding the role of SIRT1 in IBD [89,91,157,158] and how intestinal epithelial SIRT1 mediates complex environment-host interactions to regulate the integrity of the intestinal epithelium. IBD inhibits the NAD^+^/sirtuin axis as an NAD^+^ precursor supplementation and sirtuin activation slows intestinal inflammation [90]. SIRT2,3,5,6 protects against IBD [85,86,87,88,89,90], whereas SIRT1 causes it [91]. SIRT1 inhibits autophagy [159] and is lower in IBD biopsies and IL-10-deficient mice [89]. SIRT2 deletion promotes inflammatory responses by increasing NF-κB acetylation and reducing the M2-associated anti-inflammatory pathway in DSS mice [85]. Therefore, mice with DSS-induced colitis benefit from SIRT1 activation or SIRT6 overexpression [88,160,161]. Other studies have linked SIRT1 to the regulation of gut microbiota. Data from several studies show that peneth and goblet cells increase in gut-specific knockout SIRT1 mice, alleviating colitis and preventing CRC by remodeling the gut microbiota [91]. While other studies revealed the protective efft of SIRT1 in the gut against intestinal inflammation by mediating host-microbiota symbiosis [90,162], another study showed that gut epithelial SIRT1-deficient DSS mice had higher fecal bile acid concentrations, altering gut microbial composition and worsening colitis [90]. Intestinal SIRT1 plays a protective role against intestinal inflammation by facilitating host-microbiota symbiosis. It accomplishes this by regulating the gut microbiota through the modulation of bile acid metabolism, which in turn impacts gut inflammation and susceptibility to IBD [93]. Loss of intestinal epithelial SIRT1 results in inflammatory damage of the gut which is heavily influenced by the gut microbiota [163]. Since activating SIRT1 could prevent and treat experimental colitis in mice, it could be used to treat IBD in humans [90].

#### 4.2.2. CD38

CD38, a type II glycosylated membrane protein, catalyzes the production of circulating ADP-ribose from NAD^+^ and is involved in T-cell activation and proliferation [94]. CD38 is a multifunctional enzyme that converts NAD^+^ into ADPr (NAD^+^ glycohydrolase activity) and cADPr (ADPr cyclase activity) and hydrolyzes cADPr to ADPr (cADPr hydrolase activity) [95,96]. In high concentrations of free pyridine, the enzyme catalyzes a base exchange reaction (transglycosylation activity) to form nicotinic acid adenine dinucleotide phosphate (NAADP) from NADP^+^. Both cADPr and NAADP are potent intracellular Ca^2+^ mobilizers [101] and they are bound by different receptors targeting different Ca^2+^ stores. It has been shown that cADPr mobilizes ER Ca^2+^ stores via ER calcium channel receptors [164,165], whereas NAADP releases Ca^2+^ from lysosomes and the ER [166].

CD38 inhibition has emerged as a promising strategy for increasing cellular NAD^+^ levels. Cellular NAD^+^ levels can increase significantly in response to low-level CD38 inhibition. Inhibition of CD38 with flavonoid epiprotein increased NAD^+^ by 50%, similar to 300 mg NR [97,98]. The CD38 inhibitor 78c reversed the age-related decline in NAD^+^ levels and improved glucose tolerance, muscle function, exercise capacity, and cardiac function in mouse models of natural and accelerated aging [97,102]. In addition, aged wild-type mice had about half the NAD^+^ of young mice, while CD38-knockout in aged mice maintained their NAD^+^ levels and resisted the negative effects of a high-fat diet, such as hepatic lipidosis and glucose intolerance. These results show that inhibiting CD38 delays aging and improves metabolic fitness [97,103,167,168].

CD38 is involved in several inflammatory processes. CD38 interacts with other proteins to form a signaling complex that, as an immune cell receptor, regulates cell adhesion, differentiation, and proliferation [169]. The expression of CD38 increases in response to cytokine, interferon, and endotoxin stimulation, and contributes to the pro-inflammatory phenotype of innate immune cells [99]. CD38 also regulates the production of T lymphocytes [167]. Inflammation causes NAD^+^ loss via the expression of CD38 in pro-inflammatory M1-like macrophages [170]. Senescence-associated secretory phenotype (SASP) promotes macrophage proliferation and CD38 expression [170]. CD38-deficient mice have an impaired humoral immune response, development of regulatory T-cells, neutrophil chemotaxis, and dendritic cell trafficking and are more susceptible to bacterial infection [99].

Numerous studies have shown that CD38 activity may aggravate IBD [104]. First, IBD in humans and colitis in mice express CD38 in colon mucosal residents and infiltrating immune cells [58,104]. Regulatory T cells in the peripheral blood of active IBD patients and intestinal lamina propria T lymphocytes express CD38 [105,171]. Schneider et al. showed that CD38 deficiency decreases immune cell infiltration and improves DSS-induced colitis [106]. CD38^−/−^ mice have milder colitis, and CD38-targeted cancer and aging therapies may also result in IBD [103,104,172]. CD38 can regulate IBD via NAD^+^ metabolism. In the gut tissues of IBD patients, proteins such as CD38 involved in NAD^+^ metabolism are upregulated, suggesting that the NADase activity of CD38 increases NAD^+^ levels. The CD38 protein is abundant in the inflamed intestinal mucosa of UC patients and colocalizes with the macrophage marker F4/80 [58]. These findings suggest that CD38 may regulate NAD^+^ during IBD, but how or if its activity as an ADPr cyclase or cADPr hydrolase affects disease progression is unclear. CD38 receptor activity may modulate cellular responses to induce inflammation. However, more research is needed to determine how CD38 drives gut inflammation.

#### 4.2.3. PARP

PARP is a ribozyme that decomposes NAD^+^. Poly-ADP-ribosylation occurs when the enzyme breaks the nicotinamide-glycosidic bond of NAD^+^ to create ADPr polymers. PARP contains 17 enzymes, 16 of which transfer ADP ribose from NAD^+^ to macromolecular targets (proteins, DNA, and RNA) in humans [114].

In cell experiments with genotoxic drugs that damage DNA, increasing PARP activity decreases NAD^+^ levels. PARP activation reduces NAD^+^ levels by 10–20% within 5–15 min [115,116]. PARP1 inhibitors reduce inflammation and proliferation in peritonitis, septic shock, and ovarian cancer [120]. PARP inhibitors reduce DNA-induced NAD^+^ loss in human neuroblastoma cells that lack DNA repair [173]. In mice with early alcoholic steatohepatitis, PARP inhibition increased hepatic NAD^+^ levels and improved metabolism, inflammation, and oxidative stress by activating SIRT1 [117,118]. On the other hand, in PARP1-KO mice, NAD^+^ levels in brown adipose tissue and skeletal muscle increased by 100% and 50%, respectively, which improved the mitochondrial function [174,175]. PARP inhibitors have demonstrated the ability to extend the lifespan in CS models and alleviate the pronounced aging phenotype resulting from PARP1 overactivation in XDP models. In XDP models, characterized by sustained PARP activation and NAD^+^ decline, PARP inhibitors effectively mitigated these effects [176,177].

PARP1 co-activates the pro-inflammatory transcription factor NF-κB to cause inflammation in many inflammatory diseases [119]. PARP1 knockout mice are resistant to LPS and have reduced NF-κB-dependent pro-inflammatory gene expression [178]. Inflammation produces reactive oxygen and nitrogen, such as nitric oxide, which is linked to CD and UC [179,180]. Reactive oxygen and nitrogen damage DNA and activate PARP1 [181]. PARP1 and PARP2 cause colitis in mice [121,122,179,180,181]. PARP1-deficient mice prevented DSS-induced acute mucosal injury, inflammation, and death [121]. Overactivated PARP1, depleted NAD^+^, downregulated SIRT1, and depleted SIRT1 caused mucosal atrophy in IBD patients [123]. IBD patients and partial CRCs have an inflammatory microenvironment that strongly promotes malignancy [182]. PARP1 knockout mice had lower intestinal inflammation and fewer tumors than WT mice with AOM/DSS-induced colorectal cancer [183]. In rats with necrotizing enterocolitis, PARP1 inhibition by NAM reduced intestinal injury [184]. These results show that PARP1 drives colitis, and its inhibition effectively alleviates IBD.

#### 4.2.4. NAMPT and NAPRT

Nicotinamide phosphoribosyl transferase (NAMPT) and nicotinate phosphoribosyl transferase (NAPRT) are two intracellular enzymes that catalyze the first step in the biosynthesis of NAD^+^ from nicotinamide and nicotinic acid (NA), respectively. NAMPT, also known as B-cell pre-colony enhancer or visfatin [107], has cytokine-like functions that enhance B-cell pre-colonies, monocyte growth, and macrophage survival [185,186,187]. NAPRT, a homologous enzyme to NAMPT, has also been observed to be released by cells, contributing to an enhanced inflammatory response. However, it remains uncertain whether these two enzymes share the same receptors and mechanisms [188,189,190,191]. By precisely modulating intracellular NAD^+^ levels, both enzymes play crucial roles in regulating and reprogramming cellular metabolism, as well as influencing the activity of NAD^+^-dependent enzymes, such as sirtuins, PARPs, and NADases.

Intracellular NAMPT is the rate-limiting enzyme in the NAD^+^ salvage pathway. Upregulation of NAMPT increases NAD^+^, catalyzes the conversion of NAM to NMN, the precursor to NAD^+^, and counteracts NAD^+^ turnover caused by NAD^+^ decomposition. This ensures the supply of NAD^+^ and catalyzes the rate-limiting step of the NAD^+^ remediation pathway [40]. Several studies have reported that NAPMT is expressed in many cell types and involves many biological processes, including cellular energy metabolism, circadian rhythms, and immunity [107]. Extracellular NAMPT (eNAMPT) is secreted by various cell types, including neutrophils, microglia, macrophages, and fat cells [40]. Secretion of eNAMPT is triggered by cellular stress, nutritional environment, and inflammatory signals. Once secreted, this protein activates intracellular signaling pathways in various cell types, including immune cells, fat cells, and cancer cells [192]. Extracellular NAMPT is proliferative, anti-apoptotic, pro-inflammatory, angiogenic, and metastatic.

NAPRT catalyzes the conversion of NA and PRPP into nicotinic acid mononucleotide (NAMN) and pyrophosphate (PPi). Originally named NAMN pyrophosphorylase, this enzyme was first described by Handler in human erythrocytes, where it increased NAD^+^ levels [193]. NAPRT activity exhibits a more tissue-specific distribution, with enzyme activity being detected in various mouse tissues [12]. Notably, in the mouse liver, intestine, heart, and kidney, Na (nicotinic acid) serves as a more efficacious precursor than Nam (nicotinamide) for NAPRT activity [194]. Unlike NAMPT, NAPRT is not inhibited by NAD^+^, which explains its significantly higher efficiency in increasing NAD^+^ levels in vivo [195,196]. Additionally, NAPRT is strongly activated by phosphate, whereas ATP acts as an allosteric modulator of the enzyme [193,197,198].

Throughout the course of evolution, both NAMPT and NAPRT have acquired novel roles as extracellular endogenous mediators of inflammation. Mounting evidence suggests that extracellular (e)NAMPT and eNAPRT belong to a distinct class of soluble factors with effects akin to cytokines, lipoproteins, and damage-associated molecular patterns (DAMPs). Elevated levels of eNAMPT have been documented in various metabolic and inflammatory disorders, such as obesity, diabetes, and cancer. On the other hand, eNAPRT is emerging as a promising biomarker for sepsis and septic shock. Studies have shown that serum eNAMPT is increased in both CD and UC and may be associated with the disease stage [62,199]. In a recent cohort study of IBD patients, eNAMPT and eNAPRT levels were evaluated and found to be elevated in the blood and feces of IBD patients, and their levels correlated with pathological scores and high-sensitivity C-reactive protein (hsCRP) and confirmed the representation of predictive biomarkers for anti-TNF therapy response [200].

Overactivation of NAMPT promotes tumor growth and gut pro-inflammatory cytokines [18]. FK866, a small NAMPT inhibitor, reduces mucosal NAD^+^ and NAD^+^-dependent enzymes (including PARP1, SIRT6, and CD38), NF-κB pathway activation, and inflammatory cell infiltration, thereby improving DSS-induced colitis in mice and preventing inflammation-related tumors [18]. Other studies have shown that the pharmacological inhibition of NAMPT with FK866 (APO866) reduces cellular levels of ATP and impairs the in vitro secretion of pro-inflammatory mediators such as IL-1, IL-6, and TNF-α. NAMPT inhibition is consistent with the effects of NAD^+^ depletion, NAD^+^ decomposition enzyme inhibition, and cytokine release inhibition and is a promising target for treating IBD [18]. The development of a new NAPRTi should also be considered. Previous studies have shown that structural analogs of NA are able to inhibit NAPRT enzyme activity [193,196]. Among such compounds, 2-hydroxyoctanoic acid (2-HNA) is the most promising, exhibiting significant inhibition of NAPRT enzyme activity and function in in vitro ovarian cancer and xenograft models [201]. The use of NAMPT and NAPRT inhibitors appears to be a promising strategy for the treatment of IBD [18].

#### 4.2.5. NNMT

NNMT, a cytoplasmic metabolic enzyme, catalyzes the n-methylation of nicotinamide with s-adenosyl-1-methionine as the methyl donor, producing methyl nicotinamide (MNA) and releasing SAH [129,202,203,204]. Although NNMT is mostly expressed in the liver, it is also found in the brain, kidney, adipose tissue, endothelium, thyroid, pancreas, and intestinal tissues [202,205,206,207,208,209].

Studies have reported that NNMT is essential for niacin and nicotinamide metabolism. NNMT methylates and excretes Nam, thereby reducing NAD^+^ precursors [41]. Surprisingly, NNMT showed a completely different function during NAD^+^ metabolism than Nam removal alone. Most NAD^+^-decomposing enzymes are inhibited by Nam. It has been experimentally demonstrated that NNMT can remove excess Nam and maintain high levels of pro-inflammatory signaling and continuous transmission [124]. Inhibiting NNMT increases NAD^+^ concentrations because NAM is not degraded but completely reconverted to NAD^+^ [47]. NAD^+^ supplementation aids mitochondrial homeostasis [210].

Studies have shown that abnormal NNMT expression may influence tumor development, invasion, and metastasis [206,211,212]. NNMT is a prognostic marker for early-stage colorectal cancer [213] and guides adjuvant chemotherapy. In breast, esophageal, colorectal, and melanoma cancers, NNMT overexpression reduces drug sensitivity and increases chemotherapy resistance [125,126,127,128]. In addition, NNMT promotes metabolic plasticity, epigenetic reprogramming, and NAD^+^ depletion, thereby preventing tumor cell senescence and chemosensitivity [126].

Accumulating evidence shows that NNMT regulates histone methylation, polyamine flux, and NAD^+^-dependent SIRT1 signaling, making it a novel target for treating obesity and type 2 diabetes [47]. Furthermore, NNMT knockdown increases energy expenditure and protects cells from diet-induced obesity [131]. Hong et al. found that NNMT, a positive regulator of gluconeogenesis, stabilizes the SIRT1 protein in hepatocytes to promote glucose metabolism [129]. Therefore, NNMT could be essential for glucose metabolism. Small-molecule NNMT inhibitors activate aging muscle stem cells and enhance skeletal muscle regeneration [214].

Recent studies have linked NNMT to inflammation. Patients with chronic obstructive pulmonary disease (COPD) with muscle wasting have higher lung and skeletal muscle NNMT levels [133]. In addition, concanavalin A-induced experimental liver injury and pulmonary arterial hypertension models show increased NNMT expression and activity [134,135]. Human skeletal muscle myoblasts directly stimulated by IL-6, TNF-α, and TGF-β with an increase in NNMT expression suggest that increased inflammation is the cause and serves as a protective compensatory response to inflammatory injury [132,215,216]. The role of NNMT in inflammatory diseases needs further study. The liver of HFD mice overexpressing NNMT expressed more IL-1b, TNF-α, F4/80, and CD68 [217]. More recently, it has been demonstrated that NNMT deficiency reduces kidney inflammation by increasing NAD^+^ and SIRT1 levels and decreasing NF-κB acetylation. Mice with early nephritis from NNMT-KO had significantly lower levels of IL-1b and F4/80-positive cells and increased SIRT1 and SIRT7, inhibiting tissue inflammation via NF-κB, suggesting that SIRT1-mediated NF-κB deacetylation participates in the mechanism by which NNMT inhibition improves fibrosis [130].

There is limited evidence in the literature linking NNMT to IBD. The NAD^+^ pathway transcriptome reconstruction shows that IBD increases salvage biosynthesis and NAD^+^ utilization. IBD patients treated with infliximab had normalized NNMT and NAD^+^ levels. Treatment-related NAD^+^ levels affect NNMT expression [124]. However, more research is needed to elucidate the expression, role, and mechanism of NNMT in IBD.

### 4.3. IBD and NAD^+^ Regulation

#### 4.3.1. NAD^+^ and Mitochondrial Dysfunction

Mitochondria regulate cell metabolism and viability and maintain cell integrity and function [133]. Recent research has shown that mitochondria are essential for coordinating innate and adaptive immune responses. Inflammation can begin with mitochondrial dysfunction and ROS production [218]. Elevated ROS levels in the gut activate inflammatory and cell death pathways [219]. Therefore, targeting ROS in cells could reduce damage to the gut barrier caused by inflammation.

The process of NAD^+^ metabolism is intricately intertwined with mitochondrial function. NAD^+^ serves as a critical intermediate in cellular metabolism and acts as an enzymatic cofactor in redox reactions, including glycolysis, the tricarboxylic acid (TCA) cycle, and fatty acid oxidation (FAO) [6]. These reactions produce NADH, an electron donor from the nicotinamide of NAD^+^ that synthesizes ATP via mitochondrial oxidative phosphorylation [9]. Mitochondrial function and energy substitution depend on the NAD^+^/NADH ratio, regulated by mitochondrial electron flux [36]. Reduced NAD^+^ levels impair filamentous cell activity, epigenetic chromatin structure [220], mitochondrial metabolism, oxidative stress, and ATP production, thereby promoting inflammation and cellular damage [221]. Cameron et al. found that LPS induction activated mitochondrial ROS production, leading to DNA damage, PARP activation, and NAD^+^ depletion in macrophages [113].

Mitochondrial dysfunction is linked to defects in NAD^+^ metabolism. NAD^+^ supplementation improves mitochondrial performance and reduces mitochondrial damage and ROS production [46,177,222]. The NAD^+^ precursor NAM restores the NAD^+^/NADH balance and reduces IFB-γ production and Th1 differentiation in vitro [223]. Providing NAD^+^ precursors and targeting NAD^+^ biosynthesis/degradation enzymes could reverse mitochondrial dysfunction. This suggests that NAD^+^ metabolism plays a role in regulating mitochondrial function [6,224]. Minhas et al. found that macrophages synthesize NAD^+^ via the kynurenine pathway. The authors also reported that genetic ablation (in Ido^−/−^ and Qprt^−/−^ mice) or pharmacological disruption (1-methyl-L-tryptophan and phthalic acid) reduced intracellular NAD^+^ concentrations, impairing mitochondrial respiration and increasing glycolysis in vitro [225]. These metabolic changes increase CD86 and CD64 expression, decrease CD206 and CD23 expression, and impair phagocytosis [225]. The exogenous NAD^+^ precursor NMN restored mitochondrial respiratory parameters and pro-inflammatory markers [19]. NAD^+^ levels also regulate mitochondrial metabolism via sirtuins [66]. Low NAD^+^ levels decrease SIRT1 and SIRT3 activity, decrease vital mitochondrial activity, alter mitochondrial morphology, and hyperacetylate mitochondrial proteins [102,168,226,227]. Hyperactivated PARP1 reduces mitophagy due to SIRT1 impairment [177].

Mitochondrial dysfunction has been linked to NAD^+^ deficiency. NADH, the reduced form of NAD^+^, is oxidized back to NAD^+^ in complex I of the mitochondrial electron transport chain (ETC) and provides metabolic energy [6]. In addition, mitochondrial dysfunction decreases NAD^+^/NADH ratio and impairs SIRT3 activity [227]. The NAD^+^/NADH ratio is imbalanced in CD4+ T cells lacking mitochondrial transcription factor A (Tfam) that controls mitochondrial DNA expression. To compensate for mitochondrial dysfunction, CD4+ T cells lacking Tfam switch to glycolysis, decrease NAD^+^, increase the pro-inflammatory Th1 phenotype, secrete IFB-Γ and TNF-α, and inhibit IL-10 [228]. Linezolid, a ribosome-targeting antibiotic, affects the mitosome function and cell electron transport chain of Th17 cells. Mitochondrial respiration impairs NAD^+^ regeneration, lowering the NAD^+^/NADH ratio and decreasing Th17 effector function [229].

Understanding the relationship between NAD^+^ and mitochondria could help explain the pathophysiology of IBD. Inflammatory tissues have higher levels of NAM and ADR and lower levels of NAD^+^. Mitochondrial status and NAD^+^ metabolism are interdependent, and changes in the organism affect inflammation. Mitochondrial dysfunction is a major cause of IBD pathogenesis [230,231,232]. The intestinal mucosa of IBD is characterized by hypoxia and increased oxidative stress implicated in various genes involved in mitochondrial function, such as CUL2, LACC1, and NADPH oxidase [233,234,235,236,237]. A recent metabolic analysis showed NAD^+^ metabolic dysregulation and altered mitochondrial status in UC patients. The NAD^+^/NAM ratio decreased in patients with active UC, distinguishing the degree of inflammation from UC. UC alters mitochondria, resulting in a lower mitochondrial density and number in colon cells [17]. These findings suggest a link between mitochondrial dysfunction and inflammation in UC and NAD^+^ metabolism.

#### 4.3.2. Intestinal Epithelial Barrier

The intestinal epithelium forms a selective barrier that blocks toxicants and microbes from the lumen but allows nutrient absorption [238]. The intestinal epithelial barrier relies on the tight junction (TJ), a circumferential protein complex at opposing apical/basolateral cell junctions [239]. The occludin and claudin transmembrane protein families form the TJ [240] and prevent paracellular transport [241]. Inflammatory diseases, such as IBD, cholestasis, hemorrhagic shock, and sepsis, damage the intestinal epithelial barrier [242].

Extracellular NAD^+^ prevented activation, induced nitric oxide synthase, increased NO production, and improved epithelial permeability in inflammatory epithelial cells [243]. NAD^+^ improved intestinal mucosal permeability in LPS-induced CACO-2 cells [60], indicating that NAD^+^ can reduce the structural and functional changes in pro-inflammatory intestinal epithelial cells. Another study found that the overexpression of SIRT1 inhibited LPS-induced pro-inflammatory cytokines (IL-6, IL-8, and TNF-α), impaired the intestinal epithelial barrier, and reduced the inflammatory response and intestinal epithelial barrier dysfunction [244]. Quinone oxidoreductase 1 (NQO1) reduces quinone metabolites using NADH as an electron donor [245,246], regulating NAD and NADH in various cellular systems. Quinone oxidoreductase, also known as the antioxidant flavocyanin [247], clears ROS. NQO1 promotes the barrier function of the intestinal epithelium in mice by regulating the transcription of tight junction molecules. A lack of NQO1 can exacerbate colon inflammation [248]. As mentioned above, the intestinal cells have NAD^+^ receptors. These receptors could be drug targets to treat intestinal epithelium in an inflammatory environment.

#### 4.3.3. Intestinal Stem Cells

Adult stem cells use glycolysis as an energy source to avoid oxidative stress pathways during mitochondrial respiration [249]. However, mitochondrial defects are a common cause of adult stem cell senescence, as oxidative respiration is essential for their function in old age [250]. Early aging mediated by DNA repair defects degrades NAD^+^ through PARP and the loss of mitochondrial homeostasis, reducing MuSC numbers and self-renewal [177]. The activation of NAD^+^ and SIRT1 can repair mitochondrial defects in aging stem cells and DNA repair-deficient cells. Reduced SIRT3 or SIRT7 activity in hematopoietic stem cells impairs the regenerative capacity of aged mouse hematopoietic stem cells (HSCs) [251,252]. Muscle stem cells (MuSC) have lower NAD^+^ levels and SIRT1 activity with age, contributing to the decline in NAD^+^ [214]. NR, an NAD^+^ precursor, improves muscle, neural, and melanocyte stem cell function in aged mice, rejuvenating MuSCs and extending lifespan [253].

The intestinal epithelium is rapidly renewed by the ISC. Early ISC aging research focused on the intestinal epithelium of fruit flies [254]. Drosophila gut stem cells proliferate with age due to environmental changes or tissue damage. Mammalian ISCs are mainly Lgr5-expressing cells at crypt bases [255,256]. Recent studies have reported a decline in ISC function in mammals with advancing age, thereby highlighting the impact of aging on ISC dynamics. Interestingly, it has been observed that the modulation of Wnt signaling pathways can ameliorate the impaired ISC function commonly observed in older individuals [257,258]. Paneth cells support ISCs, regardless of age. In contrast, ISCs cells in mice become less active with age [259]. NAD^+^ supplementation with precursor NR can repair age-related ISC deficiencies and restore ISC quantity and vitality [62]. Compared to young mice, NR treatment reduced the sensitivity of aged mice to DSS, suggesting that NR can repair the damage in the gut of old mice by restoring the ISC pool [62]. Therefore, increasing NAD^+^ levels can activate ISCs in the intestine, speeding up intestinal barrier repair and promoting the recovery of IBD mucosa.

## 5. IBD NAD^+^ Regulation: Clinical Possibility

Cellular NAD^+^ can potentially prevent or treat many diseases. Strategies to regulate NAD^+^ concentrations have been demonstrated in a variety of animal models, including obesity, fatty liver, myocardial hypertrophy/ischemia, acute kidney injury, muscular dystrophy, mitochondrial myopathy, type II diabetes, hearing loss, and neurodegeneration [210]. Further research is needed to translate these effects into clinical usage interventions.

The metabolism of NAD^+^ during the immune response is intricate, and its diverse supplementation approaches (such as using precursors and biosynthetic activation) and interference with various stages (such as activation and effector phases) may yield distinct outcomes [260]. NAD^+^ precursors, such as tryptophan (Trp), NA, NMN, and NR, can increase NAD^+^ levels by inhibiting PARP1 and CD38, regulating biosynthesis, or increasing bioavailability [36].

Several studies have demonstrated that strategies that regulate NAD^+^ production and the breakdown pathway can efficiently treat IBD (Table 2). In DSS mice, NAD^+^ precursor NMN improved inflammatory status in the gut, colon length recovery, barrier function, and serum pro-inflammatory factor expression; and increased beneficial bacteria Firfiricutes, Verrucomicrobia, Akkermansia, and Lactobacillus, whereas decreasing Bacteroidetes. NMN reduced intestinal mucosal permeability and restored gut microbiota composition and function. NMN administration activated the NAMPT-dependent NAD^+^ biosynthetic pathway, increased NAD^+^ content, and inhibited DSS-induced colitis disease severity in mice, suggesting that NMN might be a new therapeutic strategy for IBD [261]. In TNBS-induced colitis mice, resveratrol administration upregulated SIRT1, activated the antioxidant program related to NF-E2-related factor (NRF2), and inhibited NF-κB signaling [262]. Elsewhere, it has been reported that Cay10591 activates SIRT1 and inhibits NF-κB signaling and inflammatory cytokine production in TNBS or oxazolidinone-induced mice colitis [89]. Catalpol reduces endoplasmic reticulum stress and upregulates SIRT1 in TNBS-induced colitis animals [263]. EX-527 and Nolisoboldin suppressed SIRT1 expression and induced Treg differentiation in DSS-induced colitis mice [65,264]. PJ34 reduces intestinal inflammation in mice with DSS colitis by inhibiting PARP1 [265]. 1,5-Dihydroxyisoquinoline can inhibit PARP1 and NF-κB/AP-1 [266,267] and FK866 can suppress NAMPT, thereby reducing mucosal immunity in intestinal isolated lamina propria monocytes (LPMNC) and DSS mice in IBD patients and cytokine release [18]. In acute UC patients, cyclosporine A inhibits SIRT6 expression, peripheral blood neutrophil function, and migration [268]. A study that analyzed gene expression data from patients with IBD and reconstructed metabolic pathways identified tryptophan metabolism as a pathway associated with the disease and its treatment. Additionally, the study found that NNMT can be a promising drug target for IBD, as it may help restore mucosal NAD^+^ and related metabolites [159].

The management of IBD commonly entails the utilization of immune-related medications. However, this approach carries the inherent risk of heightened susceptibility to infections, particularly when employing biologics for immunosuppressive therapy in patients with IBD [270]. Over the past century, numerous studies have revealed a connection between NAD^+^ and infection, shedding light on this intriguing relationship. Murray et al. reported that HIV-1 infection in human cells reduces intracellular NAD^+^ levels and inhibits the activity of NAD^+^ on PARP enzymes. This change can be reversed by using NAD^+^ [271]. Similarly, due to specific immune regulatory changes in HLA-DR expression, several scientists suggested using NAD^+^ to treat tuberculosis [272,273]. Hence, it can be inferred that the administration of immunobiology capable of elevating NAD^+^ levels may hold promise in mitigating the risk of infections associated with conventional immunosuppressive agents. This insight offers valuable guidance in the development of novel drug therapies for IBD.

## 6. Conclusions

Ongoing research is gradually unraveling the immunometabolism of NAD^+^, bringing forth new exciting discoveries. The treatment of IBD through the manipulation of NAD^+^-related metabolic pathways presents numerous unanswered questions that require further exploration in the times ahead. For instance, further research is needed to better understand the pharmacokinetics of NAD^+^ and its metabolic enzymes and metabolites in vivo as well as its various therapeutic effects at different stages of IBD. Second, does NAD^+^, as an important factor influencing metabolism, have a specific effect on the gut microbiota, and does it regulate the progression of intestinal inflammation? Finally, given that the gut contains many complex cell types, does NAD^+^ have complementary or antagonistic effects on different cell types? Furthermore, given the therapeutic role of NAD^+^ precursors and NAD^+^-related metabolic enzymes in various diseases, it is critical to fully explore their utility in IBD patients and investigate the therapeutic doses and routes of administration with minimal side effects.

## Figures and Tables

**Table 1:** NAD^+^ metabolic enzymes and IBD.

NAD^+^ Metabolic Enzyme	Intracellular or Extracellular	Regulation of NAD^+^	Cellular Processes and Pathways	Illnesses	IBD-Related Studies	Mechanisms Associated with IBD
Sirtuins	Intracellular	Sirtuins are NAD^+^ substrates and cofactors in deacetylated ADP-ribosylation reactions. In chronic inflammatory diseases, NAD^+^ and SIRT are downregulated in specific tissues [66].	Energy shifts [67], cell differentiation [68], apoptosis [69], autophagy [70], development [71], and metabolism [72]; regulates circadian clock proteins CLOCK/Per [73,74,75,76,77], NF-κB/p65 [78], PPAR-γ/pgc1α [79], NLRP3 inflammation [80], TLR4 [81].	Fat deposits in obesity and inflammation, atherosclerosis [82], Alzheimer’s [83], and sepsis [84].	Some sirtuins (SIRT2, 3, 5, 6) protect against IBD [85,86,87,88,89,90] and some sirtuins (SIRT1) might be pathogenic [91].	Activate macrophages [85,92] and remodel the gut microbiota [90,93,94].
CD38	Intracellular and extracellular	Type II glycosylated membrane protein CD38 converts NAD^+^ to ADPr and cADPr and hydrolyzes cADPr to ADPr [95,96]. Low-level CD38 inhibition increases cellular NAD^+^ levels [97,98].	Regulates cell adhesion, differentiation, and proliferation [99]; mobilizes intracellular Ca^2+^ [100,101]; improves glucose metabolism and maintains lipid homeostasis [97,102]; repels neutrophils [99].	Aging [103].	Potential pathogenicity in IBD [58,104,105,106]	Activation and proliferation of T cells [105].
NAMPT	Intracellular and extracellular	NAMPT upregulation increases NAD^+^, and the precursor NMN that converts NAM to NAD^+^ is essential for cellular NAD^+^ supply and rate-limiting steps in the NAD^+^ remediation pathway [40].	Regulates energy metabolism, circadian rhythm, and immunity production [107]; proliferation, anti-apoptotic, pro-inflammatory, pro-angiogenic, and metastatic properties [108].	Sepsis, rheumatoid arthritis, diabetes [109,110,111].	NAMPT inhibition prevents experimental colitis in humans and mice [18,62] and colon tumor pathogenesis in mice [18,112].	Reduces NF-κB activation and inflammatory cell infiltration [18]; decreases cell ATP levels; inhibits IL-1β, IL-6, and TNF-α secretion in vitro [113].
PARP	Intracellular	NAD^+^ decomposing enzyme, cleavage of the nicotinamide-glycosidic bond of NAD^+^ to generate ADPr polymer [114]; enhances PARP activity and has significant harmful effects on NAD^+^ pools [115,116].	Pro-inflammatory, pro-proliferative, oxidative stress [117,118]; NF-κB pathway [119].	Peritonitis, septic shock, ovarian cancer [120], and alcoholic fatty liver [117,118].	PARP1 and PARP2 promote colitis in mice [121,122].	Downregulates SIRT1 and causes mucosal atrophy [123].
NNMT	Intracellular	NAD^+^-dependent pro-inflammatory signals are maintained by methylation and excretion of NAM, which reduces precursors for NAD^+^ synthesis [41,124].	Modulates tumor resistance and chemotherapy sensitivity [125,126,127,128], glucose metabolism [129]; NF-κB pathway [130].	Colorectal cancer, breast cancer, esophageal squamous cell carcinoma, colorectal cancer, melanoma [125,126,127,128], type 2 diabetes [47], obesity [131], COPD [132,133], liver injury [134,135].	Infliximab-treated IBD patients show normalized NNMT expression [124].	No studies were found.

**Table 2:** Studies on NAD^+^ metabolic pathway-related medications to reduce intestinal inflammation.

Drugs	Effects on NAD^+^ Metabolic Pathways	Research Models	Mechanism	References
NMN	NAD^+^ precursor, activate the NAMPT-dependent NAD^+^ biosynthetic pathway, increase NAD^+^ content	DSS-induced colitis mice	Improves inflammatory intestine morphology, colon length, intestinal epithelial barrier, blood serum pro-inflammatory factors, and gut microbiota composition.	[261,269]
Resveratrol	Increases SIRT1 expression	TNBS-induced colitis mice	Inhibits NF-κB signaling and activates NRF2 antioxidant program.	[262]
Cay10591	Activates SIRT1	TNBS or oxazolidinone-induced colitis in mice	Block NF-κB signaling and cytokine production.	[89]
Catalpol	Activates SIRT1	TNBS-induced colitis mice	Improves endoplasmic reticulum stress	[263]
EX-527	Inhibits SIRT1	DSS-induced colitis mice	Promotes the formation of Treg	[264]
Norisoboldine	Reduce NAD^+^ levels and inhibit SIRT1	DSS-induced colitis mice	Enhances the differentiation of Treg, regulates the AhR/glycolysis axis	[65]
PJ34	Inhibits PARP1	DSS-induced colitis mice		[265]
1,5-Dihydroxyisoquinoline	Inhibits PARP1	TNBS-induced colitis rats	Inhibits NF-κB pathway and AP-1	[266,267]
FK866	NAMPT inhibitors	Isolated lamina propria mononuclear cells (LPMCs) from IBD patients and DSS-induced colitis mice	Inhibits intestinal mucosa immunity and cytokines.	[18]
Cyclosporine A	Inhibits SIRT6	Neutrophils in peripheral blood from patients with acute UC	Inhibits peripheral blood neutrophil function and migration in acute UC	[268]
NNMT	Restores NAD^+^	Intestinal mucosa from IBD patients	Tryptophan metabolism	[124]

## References

[1] Olén O., Erichsen R., Sachs M.C., Pedersen L., Halfvarson J., Askling J., Ekbom A., Sørensen H.T., Ludvigsson J.F. (2020). Colorectal Cancer in Ulcerative Colitis: A Scandinavian Population-Based Cohort Study. Lancet.

[2] Wijnands A.M., de Jong M.E., Lutgens M.W.M.D., Hoentjen F., Elias S.G., Oldenburg B. (2021). Dutch Initiative on Crohn and Colitis (ICC) Prognostic Factors for Advanced Colorectal Neoplasia in Inflammatory Bowel Disease: Systematic Review and Meta-Analysis. Gastroenterology.

[3] Zaiatz Bittencourt V., Jones F., Doherty G., Ryan E.J. (2021). Targeting Immune Cell Metabolism in the Treatment of Inflammatory Bowel Disease. Inflamm. Bowel Dis..

[4] Caruso R., Lo B.C., Núñez G. (2020). Host-Microbiota Interactions in Inflammatory Bowel Disease. Nat. Rev. Immunol..

[5] Ni J., Wu G.D., Albenberg L., Tomov V.T. (2017). Gut Microbiota and IBD: Causation or Correlation?. Nat. Rev. Gastroenterol. Hepatol..

[6] Katsyuba E., Romani M., Hofer D., Auwerx J. (2020). NAD^+^ Homeostasis in Health and Disease. Nat. Metab..

[7] Conlon N., Ford D. (2022). A Systems-Approach to NAD^+^ Restoration. Biochem. Pharmacol..

[8] Ying W. (2008). NAD^+^/NADH and NADP^+^/NADPH in Cellular Functions and Cell Death: Regulation and Biological Consequences. Antioxid. Redox Signal..

[9] Chakrabarty R.P., Chandel N.S. (2021). Mitochondria as Signaling Organelles Control Mammalian Stem Cell Fate. Cell Stem Cell.

[10] Koch-Nolte F., Haag F., Guse A.H., Lund F., Ziegler M. (2009). Emerging Roles of NAD^+^ and Its Metabolites in Cell Signaling. Sci. Signal..

[11] Cerutti R., Pirinen E., Lamperti C., Marchet S., Sauve A.A., Li W., Leoni V., Schon E.A., Dantzer F., Auwerx J. (2014). NAD^+^-Dependent Activation of Sirt1 Corrects the Phenotype in a Mouse Model of Mitochondrial Disease. Cell Metab..

[12] Zhou B., Wang D.D.-H., Qiu Y., Airhart S., Liu Y., Stempien-Otero A., O’Brien K.D., Tian R. (2020). Boosting NAD Level Suppresses Inflammatory Activation of PBMCs in Heart Failure. J. Clin. Investig..

[13] Shi Y., Wang Y., Shao C., Huang J., Gan J., Huang X., Bucci E., Piacentini M., Ippolito G., Melino G. (2020). COVID-19 Infection: The Perspectives on Immune Responses. Cell Death Differ..

[14] Jiang Y., Deng Y., Pang H., Ma T., Ye Q., Chen Q., Chen H., Hu Z., Qin C.-F., Xu Z. (2022). Treatment of SARS-CoV-2-Induced Pneumonia with NAD^+^ and NMN in Two Mouse Models. Cell Discov..

[15] Altay O., Arif M., Li X., Yang H., Aydın M., Alkurt G., Kim W., Akyol D., Zhang C., Dinler-Doganay G. (2021). Combined Metabolic Activators Accelerates Recovery in Mild-to-Moderate COVID-19. Adv. Sci. (Weinh).

[16] Adriouch S., Hubert S., Pechberty S., Koch-Nolte F., Haag F., Seman M. (2007). NAD^+^ Released during Inflammation Participates in T Cell Homeostasis by Inducing ART2-Mediated Death of Naive T Cells in Vivo. J. Immunol..

[17] Kang Y.H., Tucker S.A., Quevedo S.F., Inal A., Korzenik J.R., Haigis M.C. (2022). Metabolic Analyses Reveal Dysregulated NAD^+^ Metabolism and Altered Mitochondrial State in Ulcerative Colitis. PLoS ONE.

[18] Gerner R.R., Klepsch V., Macheiner S., Arnhard K., Adolph T.E., Grander C., Wieser V., Pfister A., Moser P., Hermann-Kleiter N. (2018). NAD Metabolism Fuels Human and Mouse Intestinal Inflammation. Gut.

[19] Navarro M.N., Gómez de las Heras M.M., Mittelbrunn M. (2022). Nicotinamide Adenine Dinucleotide Metabolism in the Immune Response, Autoimmunity and Inflammageing. Br. J. Pharmacol..

[20] Smids C., Horjus Talabur Horje C.S., Drylewicz J., Roosenboom B., Groenen M.J.M., van Koolwijk E., van Lochem E.G., Wahab P.J. (2018). Intestinal T Cell Profiling in Inflammatory Bowel Disease: Linking T Cell Subsets to Disease Activity and Disease Course. J. Crohn’s Colitis.

[21] Soto-Heredero G., Gómez de las Heras M.M., Gabandé-Rodríguez E., Oller J., Mittelbrunn M. (2020). Glycolysis—A Key Player in the Inflammatory Response. FEBS J..

[22] Diskin C., Ryan T.A.J., O’Neill L.A.J. (2021). Modification of Proteins by Metabolites in Immunity. Immunity.

[23] Mills E.L., Kelly B., O’Neill L.A.J. (2017). Mitochondria Are the Powerhouses of Immunity. Nat. Immunol..

[24] Pearce E.L., Pearce E.J. (2013). Metabolic Pathways In Immune Cell Activation And Quiescence. Immunity.

[25] Assmann N., Finlay D.K. (2016). Metabolic Regulation of Immune Responses: Therapeutic Opportunities. J. Clin. Investig..

[26] Patel C.H., Leone R.D., Horton M.R., Powell J.D. (2019). Targeting Metabolism to Regulate Immune Responses in Autoimmunity and Cancer. Nat. Rev. Drug Discov..

[27] Caviglia G.P., Dughera F., Ribaldone D.G., Rosso C., Abate M.L., Pellicano R., Bresso F., Smedile A., Saracco G.M., Astegiano M. (2019). Serum Zonulin in Patients with Inflammatory Bowel Disease: A Pilot Study. Minerva Med..

[28] de Souza H.S.P., Fiocchi C. (2016). Immunopathogenesis of IBD: Current State of the Art. Nat. Rev. Gastroenterol. Hepatol..

[29] Janney A., Powrie F., Mann E.H. (2020). Host-Microbiota Maladaptation in Colorectal Cancer. Nature.

[30] Meserve J., Facciorusso A., Holmer A.K., Annese V., Sandborn W.J., Singh S. (2021). Safety and Tolerability of Immune Checkpoint Inhibitors in Patients with Pre-Existing Inflammatory Bowel Diseases: A Systematic Review and Meta-Analysis. Aliment. Pharmacol. Ther..

[31] Larabi A., Barnich N., Nguyen H.T.T. (2020). New Insights into the Interplay between Autophagy, Gut Microbiota and Inflammatory Responses in IBD. Autophagy.

[32] Michaudel C., Sokol H. (2020). The Gut Microbiota at the Service of Immunometabolism. Cell Metab..

[33] Harden A., Young W.J., Martin C.J. (1997). The Alcoholic Ferment of Yeast-Juice. Proc. R. Soc. London. Ser. B Contain. Pap. A Biol. Character.

[34] Srivastava S. (2016). Emerging Therapeutic Roles for NAD(^+^) Metabolism in Mitochondrial and Age-Related Disorders. Clin. Transl. Med..

[35] Ansari H.R., Raghava G.P.S. (2010). Identification of NAD Interacting Residues in Proteins. BMC Bioinform..

[36] Xie N., Zhang L., Gao W., Huang C., Huber P.E., Zhou X., Li C., Shen G., Zou B. (2020). NAD^+^ Metabolism: Pathophysiologic Mechanisms and Therapeutic Potential. Signal. Transduct. Target Ther..

[37] Cantó C., Auwerx J. (2011). NAD^+^ as a Signaling Molecule Modulating Metabolism. Cold Spring Harb. Symp. Quant. Biol..

[38] Omran H.M., Almaliki M.S. (2020). Influence of NAD^+^ as an Ageing-Related Immunomodulator on COVID 19 Infection: A Hypothesis. J. Infect. Public. Health.

[39] Verdin E. (2015). NAD^+^ in Aging, Metabolism, and Neurodegeneration. Science.

[40] Burgos E.S. (2011). NAMPT in Regulated NAD Biosynthesis and Its Pivotal Role in Human Metabolism. Curr. Med. Chem..

[41] Sano A., Endo N., Takitani S. (1992). Fluorometric Assay of Rat Tissue N-Methyltransferases with Nicotinamide and Four Isomeric Methylnicotinamides. Chem. Pharm. Bull. (Tokyo).

[42] Rajman L., Chwalek K., Sinclair D.A. (2018). Therapeutic Potential of NAD-Boosting Molecules: The in Vivo Evidence. Cell Metab..

[43] Ljungberg M.C., Ali Y.O., Zhu J., Wu C.-S., Oka K., Zhai R.G., Lu H.-C. (2012). CREB-Activity and Nmnat2 Transcription Are down-Regulated Prior to Neurodegeneration, While NMNAT2 over-Expression Is Neuroprotective, in a Mouse Model of Human Tauopathy. Hum. Mol. Genet..

[44] Trammell S.A.J., Weidemann B.J., Chadda A., Yorek M.S., Holmes A., Coppey L.J., Obrosov A., Kardon R.H., Yorek M.A., Brenner C. (2016). Nicotinamide Riboside Opposes Type 2 Diabetes and Neuropathy in Mice. Sci. Rep..

[45] Yoshino J., Mills K.F., Yoon M.J., Imai S. (2011). Nicotinamide Mononucleotide, a Key NAD^+^ Intermediate, Treats the Pathophysiology of Diet- and Age-Induced Diabetes in Mice. Cell Metab..

[46] Mitchell S.J., Bernier M., Aon M.A., Cortassa S., Kim E.Y., Fang E.F., Palacios H.H., Ali A., Navas-Enamorado I., Di Francesco A. (2018). Nicotinamide Improves Aspects of Healthspan, but Not Lifespan, in Mice. Cell Metab..

[47] Kraus D., Yang Q., Kong D., Banks A.S., Zhang L., Rodgers J.T., Pirinen E., Pulinilkunnil T.C., Gong F., Wang Y. (2014). Nicotinamide N-Methyltransferase Knockdown Protects against Diet-Induced Obesity. Nature.

[48] Cantó C., Houtkooper R.H., Pirinen E., Youn D.Y., Oosterveer M.H., Cen Y., Fernandez-Marcos P.J., Yamamoto H., Andreux P.A., Cettour-Rose P. (2012). The NAD^+^ Precursor Nicotinamide Riboside Enhances Oxidative Metabolism and Protects against High-Fat Diet Induced Obesity. Cell Metab..

[49] Kannt A., Rajagopal S., Kadnur S.V., Suresh J., Bhamidipati R.K., Swaminathan S., Hallur M.S., Kristam R., Elvert R., Czech J. (2018). A Small Molecule Inhibitor of Nicotinamide N-Methyltransferase for the Treatment of Metabolic Disorders. Sci. Rep..

[50] Hsu C.-P., Oka S., Shao D., Hariharan N., Sadoshima J. (2009). Nicotinamide Phosphoribosyltransferase Regulates Cell Survival through NAD^+^ Synthesis in Cardiac Myocytes. Circ. Res..

[51] Diguet N., Trammell S.A.J., Tannous C., Deloux R., Piquereau J., Mougenot N., Gouge A., Gressette M., Manoury B., Blanc J. (2018). Nicotinamide Riboside Preserves Cardiac Function in a Mouse Model of Dilated Cardiomyopathy. Circulation.

[52] Ryu D., Zhang H., Ropelle E.R., Sorrentino V., Mázala D.A.G., Mouchiroud L., Marshall P.L., Campbell M.D., Ali A.S., Knowels G.M. (2016). NAD^+^ Repletion Improves Muscle Function in Muscular Dystrophy and Counters Global PARylation. Sci. Transl. Med..

[53] Poyan Mehr A., Tran M.T., Ralto K.M., Leaf D.E., Washco V., Messmer J., Lerner A., Kher A., Kim S.H., Khoury C.C. (2018). De Novo NAD^+^ Biosynthetic Impairment in Acute Kidney Injury in Humans. Nat. Med..

[54] Chiarugi A., Dölle C., Felici R., Ziegler M. (2012). The NAD Metabolome--a Key Determinant of Cancer Cell Biology. Nat. Rev. Cancer.

[55] Buonvicino D., Mazzola F., Zamporlini F., Resta F., Ranieri G., Camaioni E., Muzzi M., Zecchi R., Pieraccini G., Dölle C. (2018). Identification of the Nicotinamide Salvage Pathway as a New Toxification Route for Antimetabolites. Cell. Chem. Biol..

[56] Vachharajani V., Liu T., McCall C.E. (2014). Epigenetic Coordination of Acute Systemic Inflammation: Potential Therapeutic Targets. Expert Rev. Clin. Immunol..

[57] Schilling E., Wehrhahn J., Klein C., Raulien N., Ceglarek U., Hauschildt S. (2012). Inhibition of Nicotinamide Phosphoribosyltransferase Modifies LPS-Induced Inflammatory Responses of Human Monocytes. Innat. Immun..

[58] Ning L., Shan G., Sun Z., Zhang F., Xu C., Lou X., Li S., Du H., Chen H., Xu G. (2019). Quantitative Proteomic Analysis Reveals the Deregulation of Nicotinamide Adenine Dinucleotide Metabolism and CD38 in Inflammatory Bowel Disease. Biomed. Res. Int..

[59] Galli U., Colombo G., Travelli C., Tron G.C., Genazzani A.A., Grolla A.A. (2020). Recent Advances in NAMPT Inhibitors: A Novel Immunotherapic Strategy. Front. Pharmacol..

[60] Han X., Uchiyama T., Sappington P.L., Yaguchi A., Yang R., Fink M.P., Delude R.L. (2003). NAD^+^ Ameliorates Inflammation-Induced Epithelial Barrier Dysfunction in Cultured Enterocytes and Mouse Ileal Mucosa. J. Pharmacol. Exp. Ther..

[61] Shats I., Williams J.G., Liu J., Makarov M.V., Wu X., Lih F.B., Deterding L.J., Lim C., Xu X., Randall T.A. (2020). Bacteria Boost Mammalian Host NAD Metabolism by Engaging the Deamidated Biosynthesis Pathway. Cell Metab..

[62] Colombo G., Clemente N., Zito A., Bracci C., Colombo F.S., Sangaletti S., Jachetti E., Ribaldone D.G., Caviglia G.P., Pastorelli L. (2020). Neutralization of Extracellular NAMPT (Nicotinamide Phosphoribosyltransferase) Ameliorates Experimental Murine Colitis. J. Mol. Med. (Berl.).

[63] Igarashi M., Guarente L. (2016). MTORC1 and SIRT1 Cooperate to Foster Expansion of Gut Adult Stem Cells during Calorie Restriction. Cell.

[64] Uchida R., Saito Y., Nogami K., Kajiyama Y., Suzuki Y., Kawase Y., Nakaoka T., Muramatsu T., Kimura M., Saito H. (2018). Epigenetic Silencing of Lgr5 Induces Senescence of Intestinal Epithelial Organoids during the Process of Aging. NPJ Aging Mech. Dis..

[65] Lv Q., Wang K., Qiao S., Yang L., Xin Y., Dai Y., Wei Z. (2018). Norisoboldine, a Natural AhR Agonist, Promotes Treg Differentiation and Attenuates Colitis via Targeting Glycolysis and Subsequent NAD^+^/SIRT1/SUV39H1/H3K9me3 Signaling Pathway. Cell Death Dis..

[66] Covarrubias A.J., Perrone R., Grozio A., Verdin E. (2021). NAD^+^ Metabolism and Its Roles in Cellular Processes during Ageing. Nat. Rev. Mol. Cell Biol..

[67] Araki T., Sasaki Y., Milbrandt J. (2004). Increased Nuclear NAD Biosynthesis and SIRT1 Activation Prevent Axonal Degeneration. Science.

[68] Fulco M., Schiltz R.L., Iezzi S., King M.T., Zhao P., Kashiwaya Y., Hoffman E., Veech R.L., Sartorelli V. (2003). Sir2 Regulates Skeletal Muscle Differentiation as a Potential Sensor of the Redox State. Mol. Cell..

[69] Luo J., Nikolaev A.Y., Imai S., Chen D., Su F., Shiloh A., Guarente L., Gu W. (2001). Negative Control of P53 by Sir2alpha Promotes Cell Survival under Stress. Cell.

[70] Lee I.H., Cao L., Mostoslavsky R., Lombard D.B., Liu J., Bruns N.E., Tsokos M., Alt F.W., Finkel T. (2008). A Role for the NAD-Dependent Deacetylase Sirt1 in the Regulation of Autophagy. Proc. Natl. Acad. Sci. USA.

[71] Cheng H.-L., Mostoslavsky R., Saito S., Manis J.P., Gu Y., Patel P., Bronson R., Appella E., Alt F.W., Chua K.F. (2003). Developmental Defects and P53 Hyperacetylation in Sir2 Homolog (SIRT1)-Deficient Mice. Proc. Natl. Acad. Sci. USA.

[72] Li X. (2013). SIRT1 and Energy Metabolism. Acta Biochim. Biophys. Sin..

[73] Aguilar-Arnal L., Katada S., Orozco-Solis R., Sassone-Corsi P. (2015). NAD^+^-SIRT1 Control of H3K4 Trimethylation through Circadian Deacetylation of MLL1. Nat. Struct. Mol. Biol..

[74] Asher G., Gatfield D., Stratmann M., Reinke H., Dibner C., Kreppel F., Mostoslavsky R., Alt F.W., Schibler U. (2008). SIRT1 Regulates Circadian Clock Gene Expression through PER2 Deacetylation. Cell.

[75] Bellet M.M., Nakahata Y., Boudjelal M., Watts E., Mossakowska D.E., Edwards K.A., Cervantes M., Astarita G., Loh C., Ellis J.L. (2013). Pharmacological Modulation of Circadian Rhythms by Synthetic Activators of the Deacetylase SIRT1. Proc. Natl. Acad. Sci. USA.

[76] Chang H.-C., Guarente L. (2013). SIRT1 Mediates Central Circadian Control in the SCN by a Mechanism That Decays with Aging. Cell.

[77] Nakahata Y., Kaluzova M., Grimaldi B., Sahar S., Hirayama J., Chen D., Guarente L.P., Sassone-Corsi P. (2008). The NAD^+^-Dependent Deacetylase SIRT1 Modulates CLOCK-Mediated Chromatin Remodeling and Circadian Control. Cell.

[78] Yeung F., Hoberg J.E., Ramsey C.S., Keller M.D., Jones D.R., Frye R.A., Mayo M.W. (2004). Modulation of NF-ΚB-Dependent Transcription and Cell Survival by the SIRT1 Deacetylase. EMBO J..

[79] Nemoto S., Fergusson M.M., Finkel T. (2005). SIRT1 Functionally Interacts with the Metabolic Regulator and Transcriptional Coactivator PGC-1{alpha}. J. Biol. Chem..

[80] Park S., Shin J., Bae J., Han D., Park S.-R., Shin J., Lee S.K., Park H.-W. (2020). SIRT1 Alleviates LPS-Induced IL-1β Production by Suppressing NLRP3 Inflammasome Activation and ROS Production in Trophoblasts. Cells.

[81] Liu T.F., Yoza B.K., El Gazzar M., Vachharajani V.T., McCall C.E. (2011). NAD^+^-Dependent SIRT1 Deacetylase Participates in Epigenetic Reprogramming during Endotoxin Tolerance. J. Biol. Chem..

[82] Schug T.T., Li X. (2010). Surprising Sirtuin Crosstalk in the Heart. Aging (Albany N. Y.).

[83] Qin W., Yang T., Ho L., Zhao Z., Wang J., Chen L., Zhao W., Thiyagarajan M., MacGrogan D., Rodgers J.T. (2006). Neuronal SIRT1 Activation as a Novel Mechanism Underlying the Prevention of Alzheimer Disease Amyloid Neuropathology by Calorie Restriction. J. Biol. Chem..

[84] Liu T.F., Vachharajani V., Millet P., Bharadwaj M.S., Molina A.J., McCall C.E. (2015). Sequential Actions of SIRT1-RELB-SIRT3 Coordinate Nuclear-Mitochondrial Communication during Immunometabolic Adaptation to Acute Inflammation and Sepsis. J. Biol. Chem..

[85] Lo Sasso G., Menzies K.J., Mottis A., Piersigilli A., Perino A., Yamamoto H., Schoonjans K., Auwerx J. (2014). SIRT2 Deficiency Modulates Macrophage Polarization and Susceptibility to Experimental Colitis. PLoS ONE.

[86] Zhang Y., Wang X.-L., Zhou M., Kang C., Lang H.-D., Chen M.-T., Hui S.-C., Wang B., Mi M.-T. (2018). Crosstalk between Gut Microbiota and Sirtuin-3 in Colonic Inflammation and Tumorigenesis. Exp. Mol. Med..

[87] Wang F., Wang K., Xu W., Zhao S., Ye D., Wang Y., Xu Y., Zhou L., Chu Y., Zhang C. (2017). SIRT5 Desuccinylates and Activates Pyruvate Kinase M2 to Block Macrophage IL-1β Production and to Prevent DSS-Induced Colitis in Mice. Cell Rep..

[88] Liu F., Bu H.-F., Geng H., De Plaen I.G., Gao C., Wang P., Wang X., Kurowski J.A., Yang H., Qian J. (2017). Sirtuin-6 Preserves R-Spondin-1 Expression and Increases Resistance of Intestinal Epithelium to Injury in Mice. Mol. Med..

[89] Caruso R., Marafini I., Franzè E., Stolfi C., Zorzi F., Monteleone I., Caprioli F., Colantoni A., Sarra M., Sedda S. (2014). Defective Expression of SIRT1 Contributes to Sustain Inflammatory Pathways in the Gut. Mucosal. Immunol..

[90] Wellman A.S., Metukuri M.R., Kazgan N., Xu X., Xu Q., Ren N.S.X., Czopik A., Shanahan M.T., Kang A., Chen W. (2017). Intestinal Epithelial Sirtuin 1 Regulates Intestinal Inflammation during Aging in Mice by Altering the Intestinal Microbiota. Gastroenterology.

[91] Lo Sasso G., Ryu D., Mouchiroud L., Fernando S.C., Anderson C.L., Katsyuba E., Piersigilli A., Hottiger M.O., Schoonjans K., Auwerx J. (2014). Loss of Sirt1 Function Improves Intestinal Anti-Bacterial Defense and Protects from Colitis-Induced Colorectal Cancer. PLoS ONE.

[92] Yoshizaki T., Milne J.C., Imamura T., Schenk S., Sonoda N., Babendure J.L., Lu J.-C., Smith J.J., Jirousek M.R., Olefsky J.M. (2009). SIRT1 Exerts Anti-Inflammatory Effects and Improves Insulin Sensitivity in Adipocytes. Mol. Cell. Biol..

[93] Larrosa M., Yañéz-Gascón M.J., Selma M.V., González-Sarrías A., Toti S., Cerón J.J., Tomás-Barberán F., Dolara P., Espín J.C. (2009). Effect of a Low Dose of Dietary Resveratrol on Colon Microbiota, Inflammation and Tissue Damage in a DSS-Induced Colitis Rat Model. J. Agric. Food Chem..

[94] Sandoval-Montes C., Santos-Argumedo L. (2005). CD38 Is Expressed Selectively during the Activation of a Subset of Mature T Cells with Reduced Proliferation but Improved Potential to Produce Cytokines. J. Leukoc. Biol..

[95] Hogan K.A., Chini C.C.S., Chini E.N. (2019). The Multi-Faceted Ecto-Enzyme CD38: Roles in Immunomodulation, Cancer, Aging, and Metabolic Diseases. Front. Immunol..

[96] Zocchi E., Franco L., Guida L., Benatti U., Bargellesi A., Malavasi F., Lee H.C., De Flora A. (1993). A Single Protein Immunologically Identified as CD38 Displays NAD^+^ Glycohydrolase, ADP-Ribosyl Cyclase and Cyclic ADP-Ribose Hydrolase Activities at the Outer Surface of Human Erythrocytes. Biochem. Biophys. Res. Commun..

[97] Escande C., Nin V., Price N.L., Capellini V., Gomes A.P., Barbosa M.T., O’Neil L., White T.A., Sinclair D.A., Chini E.N. (2013). Flavonoid Apigenin Is an Inhibitor of the NAD^+^ Ase CD38: Implications for Cellular NAD^+^ Metabolism, Protein Acetylation, and Treatment of Metabolic Syndrome. Diabetes.

[98] Kellenberger E., Kuhn I., Schuber F., Muller-Steffner H. (2011). Flavonoids as Inhibitors of Human CD38. Bioorg. Med. Chem. Lett..

[99] Glaría E., Valledor A.F. (2020). Roles of CD38 in the Immune Response to Infection. Cells.

[100] Perraud A.-L., Fleig A., Dunn C.A., Bagley L.A., Launay P., Schmitz C., Stokes A.J., Zhu Q., Bessman M.J., Penner R. (2001). ADP-Ribose Gating of the Calcium-Permeable LTRPC2 Channel Revealed by Nudix Motif Homology. Nature.

[101] Young G.S., Choleris E., Lund F.E., Kirkland J.B. (2006). Decreased CADPR and Increased NAD^+^ in the Cd38^−/−^ Mouse. Biochem. Biophys. Res. Commun..

[102] Camacho-Pereira J., Tarragó M.G., Chini C.C.S., Nin V., Escande C., Warner G.M., Puranik A.S., Schoon R.A., Reid J.M., Galina A. (2016). CD38 Dictates Age-Related NAD Decline and Mitochondrial Dysfunction through an SIRT3-Dependent Mechanism. Cell Metab..

[103] Tarragó M.G., Chini C.C.S., Kanamori K.S., Warner G.M., Caride A., de Oliveira G.C., Rud M., Samani A., Hein K.Z., Huang R. (2018). A Potent and Specific CD38 Inhibitor Ameliorates Age-Related Metabolic Dysfunction by Reversing Tissue NAD^+^ Decline. Cell Metab..

[104] Schneider M., Schumacher V., Lischke T., Lücke K., Meyer-Schwesinger C., Velden J., Koch-Nolte F., Mittrücker H.-W. (2015). CD38 Is Expressed on Inflammatory Cells of the Intestine and Promotes Intestinal Inflammation. PLoS ONE.

[105] Joosse M.E., Menckeberg C.L., de Ruiter L.F., Raatgeep H.R.C., van Berkel L.A., Simons-Oosterhuis Y., Tindemans I., Muskens A.F.M., Hendriks R.W., Hoogenboezem R.M. (2019). Frequencies of Circulating Regulatory TIGIT+CD38+ Effector T Cells Correlate with the Course of Inflammatory Bowel Disease. Mucosal. Immunol..

[106] Mestas J., Hughes C.C.W. (2004). Of Mice and Not Men: Differences between Mouse and Human Immunology. J. Immunol..

[107] Moschen A.R., Gerner R.R., Tilg H. (2010). Pre-B Cell Colony Enhancing Factor/NAMPT/Visfatin in Inflammation and Obesity-Related Disorders. Curr. Pharm. Des..

[108] Carbone F., Liberale L., Bonaventura A., Vecchiè A., Casula M., Cea M., Monacelli F., Caffa I., Bruzzone S., Montecucco F. (2017). Regulation and Function of Extracellular Nicotinamide Phosphoribosyltransferase/Visfatin. Compr. Physiol..

[109] Jia S.H., Li Y., Parodo J., Kapus A., Fan L., Rotstein O.D., Marshall J.C. (2004). Pre–B Cell Colony–Enhancing Factor Inhibits Neutrophil Apoptosis in Experimental Inflammation and Clinical Sepsis. J. Clin. Investig..

[110] Meier F.M.P., Frommer K.W., Peters M.A., Brentano F., Lefèvre S., Schröder D., Kyburz D., Steinmeyer J., Rehart S., Gay S. (2012). Visfatin/Pre-B-Cell Colony-Enhancing Factor (PBEF), a Proinflammatory and Cell Motility-Changing Factor in Rheumatoid Arthritis. J. Biol. Chem..

[111] El-Mesallamy H.O., Kassem D.H., El-Demerdash E., Amin A.I. (2011). Vaspin and Visfatin/Nampt Are Interesting Interrelated Adipokines Playing a Role in the Pathogenesis of Type 2 Diabetes Mellitus. Metabolism.

[112] Ye C., Qi L., Li X., Wang J., Yu J., Zhou B., Guo C., Chen J., Zheng S. (2020). Targeting the NAD^+^ Salvage Pathway Suppresses APC Mutation-Driven Colorectal Cancer Growth and Wnt/β-Catenin Signaling via Increasing Axin Level. Cell Commun. Signal..

[113] Cameron A.M., Castoldi A., Sanin D.E., Flachsmann L.J., Field C.S., Puleston D.J., Kyle R.L., Patterson A.E., Hässler F., Buescher J.M. (2019). Inflammatory Macrophage Dependence on NAD^+^ Salvage Is a Consequence of Reactive Oxygen Species-Mediated DNA Damage. Nat. Immunol..

[114] Kraus W.L., Hottiger M.O. (2013). PARP-1 and Gene Regulation: Progress and Puzzles. Mol. Aspects. Med..

[115] Goodwin P.M., Lewis P.J., Davies M.I., Skidmore C.J., Shall S. (1978). The Effect of Gamma Radiation and Neocarzinostatin of NAD and ATP Levels in Mouse Leukaemia Cells. Biochim. Biophys. Acta (BBA)-Gen. Subj..

[116] Skidmore C.J., Davies M.I., Goodwin P.M., Halldorsson H., Lewis P.J., Shall S., Zia’ee A.A. (1979). The Involvement of Poly(ADP-Ribose) Polymerase in the Degradation of NAD Caused by Gamma-Radiation and N-Methyl-N-Nitrosourea. Eur. J. Biochem..

[117] Gariani K., Ryu D., Menzies K.J., Yi H.-S., Stein S., Zhang H., Perino A., Lemos V., Katsyuba E., Jha P. (2017). Inhibiting Poly ADP-Ribosylation Increases Fatty Acid Oxidation and Protects against Fatty Liver Disease. J. Hepatol..

[118] Mukhopadhyay P., Horváth B., Rajesh M., Varga Z.V., Gariani K., Ryu D., Cao Z., Holovac E., Park O., Zhou Z. (2017). PARP Inhibition Protects against Alcoholic and Non-Alcoholic Steatohepatitis. J. Hepatol..

[119] Mangerich A., Bürkle A. (2012). Pleiotropic Cellular Functions of PARP1 in Longevity and Aging: Genome Maintenance Meets Inflammation. Oxid. Med. Cell. Longev..

[120] Jagtap P., Szabó C. (2005). Poly(ADP-Ribose) Polymerase and the Therapeutic Effects of Its Inhibitors. Nat. Rev. Drug Discov..

[121] Larmonier C.B., Shehab K.W., Laubitz D., Jamwal D.R., Ghishan F.K., Kiela P.R. (2016). Transcriptional Reprogramming and Resistance to Colonic Mucosal Injury in Poly(ADP-Ribose) Polymerase 1 (PARP1)-Deficient Mice. J. Biol. Chem..

[122] Popoff I., Jijon H., Monia B., Tavernini M., Ma M., McKay R., Madsen K. (2002). Antisense Oligonucleotides to Poly(ADP-Ribose) Polymerase-2 Ameliorate Colitis in Interleukin-10-Deficient Mice. J. Pharmacol. Exp. Ther..

[123] Lucena-Cacace A., Otero-Albiol D., Jiménez-García M.P., Muñoz-Galvan S., Carnero A. (2018). NAMPT Is a Potent Oncogene in Colon Cancer Progression That Modulates Cancer Stem Cell Properties and Resistance to Therapy through Sirt1 and PARP. Clin. Cancer Res..

[124] Wnorowski A., Wnorowska S., Kurzepa J., Parada-Turska J. (2021). Alterations in Kynurenine and NAD^+^ Salvage Pathways during the Successful Treatment of Inflammatory Bowel Disease Suggest HCAR3 and NNMT as Potential Drug Targets. Int. J. Mol. Sci..

[125] Wang Y., Zeng J., Wu W., Xie S., Yu H., Li G., Zhu T., Li F., Lu J., Wang G.Y. (2019). Nicotinamide N-Methyltransferase Enhances Chemoresistance in Breast Cancer through SIRT1 Protein Stabilization. Breast Cancer Res..

[126] Cui Y., Yang D., Wang W., Zhang L., Liu H., Ma S., Guo W., Yao M., Zhang K., Li W. (2020). Nicotinamide N-Methyltransferase Decreases 5-Fluorouracil Sensitivity in Human Esophageal Squamous Cell Carcinoma through Metabolic Reprogramming and Promoting the Warburg Effect. Mol. Carcinog..

[127] Li G., Kong B., Tong Q., Li Y., Chen L., Zeng J., Yu H., Xie X., Zhang J. (2021). Vanillin Downregulates NNMT and Attenuates NNMT-related Resistance to 5-fluorouracil via ROS-induced Cell Apoptosis in Colorectal Cancer Cells. Oncol. Rep..

[128] Campagna R., Salvolini E., Pompei V., Pozzi V., Salvucci A., Molinelli E., Brisigotti V., Sartini D., Campanati A., Offidani A. (2021). Nicotinamide N-Methyltransferase Gene Silencing Enhances Chemosensitivity of Melanoma Cell Lines. Pigment. Cell Melanoma. Res..

[129] Hong S., Zhai B., Pissios P. (2018). Nicotinamide N-Methyltransferase Interacts with Enzymes of the Methionine Cycle and Regulates Methyl Donor Metabolism. Biochemistry.

[130] Takahashi R., Kanda T., Komatsu M., Itoh T., Minakuchi H., Urai H., Kuroita T., Shigaki S., Tsukamoto T., Higuchi N. (2022). The Significance of NAD + Metabolites and Nicotinamide N-Methyltransferase in Chronic Kidney Disease. Sci. Rep..

[131] Kanakkanthara A., Kurmi K., Ekstrom T.L., Hou X., Purfeerst E.R., Heinzen E.P., Correia C., Huntoon C.J., O’Brien D., Wahner Hendrickson A.E. (2019). BRCA1 Deficiency Upregulates NNMT, Which Reprograms Metabolism and Sensitizes Ovarian Cancer Cells to Mitochondrial Metabolic Targeting Agents. Cancer Res..

[132] Kim H.C., Mofarrahi M., Vassilakopoulos T., Maltais F., Sigala I., Debigare R., Bellenis I., Hussain S.N.A. (2010). Expression and Functional Significance of Nicotinamide N-Methyl Transferase in Skeletal Muscles of Patients with Chronic Obstructive Pulmonary Disease. Am. J. Respir. Crit. Care Med..

[133] Savarimuthu Francis S.M., Larsen J.E., Pavey S.J., Duhig E.E., Clarke B.E., Bowman R.V., Hayward N.K., Fong K.M., Yang I.A. (2011). Genes and Gene Ontologies Common to Airflow Obstruction and Emphysema in the Lungs of Patients with COPD. PLoS ONE.

[134] Sternak M., Khomich T.I., Jakubowski A., Szafarz M., Szczepański W., Białas M., Stojak M., Szymura-Oleksiak J., Chłopicki S. (2010). Nicotinamide N-Methyltransferase (NNMT) and 1-Methylnicotinamide (MNA) in Experimental Hepatitis Induced by Concanavalin A in the Mouse. Pharmacol. Rep..

[135] Fedorowicz A., Mateuszuk Ł., Kopec G., Skórka T., Kutryb-Zając B., Zakrzewska A., Walczak M., Jakubowski A., Łomnicka M., Słomińska E. (2016). Activation of the Nicotinamide N-Methyltransferase (NNMT)-1-Methylnicotinamide (MNA) Pathway in Pulmonary Hypertension. Respir. Res..

[136] Kida Y., Goligorsky M.S. (2016). Sirtuins, Cell Senescence, and Vascular Aging. Can. J. Cardiol..

[137] Chang H.-C., Guarente L. (2014). SIRT1 and Other Sirtuins in Metabolism. Trends. Endocrinol. Metab..

[138] Smith J.S., Brachmann C.B., Celic I., Kenna M.A., Muhammad S., Starai V.J., Avalos J.L., Escalante-Semerena J.C., Grubmeyer C., Wolberger C. (2000). A Phylogenetically Conserved NAD^+^-Dependent Protein Deacetylase Activity in the Sir2 Protein Family. Proc. Natl. Acad. Sci. USA.

[139] Tanny J.C., Dowd G.J., Huang J., Hilz H., Moazed D. (1999). An Enzymatic Activity in the Yeast Sir2 Protein That Is Essential for Gene Silencing. Cell.

[140] Byrnes K., Blessinger S., Bailey N.T., Scaife R., Liu G., Khambu B. (2022). Therapeutic Regulation of Autophagy in Hepatic Metabolism. Acta. Pharm. Sin. B.

[141] Huang Q., Su H., Qi B., Wang Y., Yan K., Wang X., Li X., Zhao D. (2021). A SIRT1 Activator, Ginsenoside Rc, Promotes Energy Metabolism in Cardiomyocytes and Neurons. J. Am. Chem. Soc..

[142] Xiong S., Salazar G., Patrushev N., Alexander R.W. (2011). FoxO1 Mediates an Autofeedback Loop Regulating SIRT1 Expression. J. Biol. Chem..

[143] Salminen A., Kauppinen A., Suuronen T., Kaarniranta K. (2008). SIRT1 Longevity Factor Suppresses NF-KappaB -Driven Immune Responses: Regulation of Aging via NF-KappaB Acetylation?. Bioessays.

[144] Serrano-Marco L., Chacón M.R., Maymó-Masip E., Barroso E., Salvadó L., Wabitsch M., Garrido-Sánchez L., Tinahones F.J., Palomer X., Vendrell J. (2012). TNF-α Inhibits PPARβ/δ Activity and SIRT1 Expression through NF-ΚB in Human Adipocytes. Biochim. Biophys. Acta.

[145] Imai S., Guarente L. (2014). NAD^+^ and Sirtuins in Aging and Disease. Trends Cell Biol..

[146] Haigis M.C., Sinclair D.A. (2010). Mammalian Sirtuins: Biological Insights and Disease Relevance. Annu. Rev. Pathol..

[147] Natoli G. (2009). When Sirtuins and NF-KappaB Collide. Cell.

[148] Preyat N., Leo O. (2013). Sirtuin Deacylases: A Molecular Link between Metabolism and Immunity. J. Leukoc. Biol..

[149] Vachharajani V.T., Liu T., Wang X., Hoth J.J., Yoza B.K., McCall C.E. (2016). Sirtuins Link Inflammation and Metabolism. J. Immunol. Res..

[150] Houtkooper R.H., Pirinen E., Auwerx J. (2012). Sirtuins as Regulators of Metabolism and Healthspan. Nat. Rev. Mol. Cell Biol..

[151] Verdin E. (2014). The Many Faces of Sirtuins: Coupling of NAD Metabolism, Sirtuins and Lifespan. Nat. Med..

[152] Chen X., Lu Y., Zhang Z., Wang J., Yang H., Liu G. (2015). Intercellular Interplay between Sirt1 Signalling and Cell Metabolism in Immune Cell Biology. Immunology.

[153] Liu T.F., Vachharajani V.T., Yoza B.K., McCall C.E. (2012). NAD^+^-Dependent Sirtuin 1 and 6 Proteins Coordinate a Switch from Glucose to Fatty Acid Oxidation during the Acute Inflammatory Response. J. Biol. Chem..

[154] Picard F., Kurtev M., Chung N., Topark-Ngarm A., Senawong T., de Oliveira R.M., Leid M., McBurney M.W., Guarente L. (2004). Sirt1 Promotes Fat Mobilization in White Adipocytes by Repressing PPAR-γ. Nature.

[155] Lin J., Handschin C., Spiegelman B.M. (2005). Metabolic Control through the PGC-1 Family of Transcription Coactivators. Cell Metab..

[156] Vandanmagsar B., Youm Y.-H., Ravussin A., Galgani J.E., Stadler K., Mynatt R.L., Ravussin E., Stephens J.M., Dixit V.D. (2011). The NALP3/NLRP3 Inflammasome Instigates Obesity-Induced Autoinflammation and Insulin Resistance. Nat. Med..

[157] Biason-Lauber A., Böni-Schnetzler M., Hubbard B.P., Bouzakri K., Brunner A., Cavelti-Weder C., Keller C., Meyer-Böni M., Meier D.T., Brorsson C. (2013). Identification of a SIRT1 Mutation in a Family with Type 1 Diabetes. Cell Metab..

[158] Melhem H., Hansmannel F., Bressenot A., Battaglia-Hsu S.-F., Billioud V., Alberto J.M., Gueant J.L., Peyrin-Biroulet L. (2016). Methyl-Deficient Diet Promotes Colitis and SIRT1-Mediated Endoplasmic Reticulum Stress. Gut.

[159] Talero E., Alcaide A., Ávila-Román J., García-Mauriño S., Vendramini-Costa D., Motilva V. (2016). Expression Patterns of Sirtuin 1-AMPK-Autophagy Pathway in Chronic Colitis and Inflammation-Associated Colon Neoplasia in IL-10-Deficient Mice. Int. Immunopharmacol..

[160] Ren M.-T., Gu M.-L., Zhou X.-X., Yu M.-S., Pan H.-H., Ji F., Ding C.-Y. (2019). Sirtuin 1 Alleviates Endoplasmic Reticulum Stress-Mediated Apoptosis of Intestinal Epithelial Cells in Ulcerative Colitis. World J. Gastroenterol..

[161] Xu K., Guo Y., Ping L., Qiu Y., Liu Q., Li Z., Wang Z. (2020). Protective Effects of SIRT6 Overexpression against DSS-Induced Colitis in Mice. Cells.

[162] Leber A., Hontecillas R., Tubau-Juni N., Zoccoli-Rodriguez V., Abedi V., Bassaganya-Riera J. (2018). NLRX1 Modulates Immunometabolic Mechanisms Controlling the Host–Gut Microbiota Interactions during Inflammatory Bowel Disease. Front. Immunol..

[163] Wu Y.-X., Yang X.-Y., Han B.-S., Hu Y.-Y., An T., Lv B.-H., Lian J., Wang T.-Y., Bao X.-L., Gao L. (2022). Naringenin Regulates Gut Microbiota and SIRT1/PGC-1ɑ Signaling Pathway in Rats with Letrozole-Induced Polycystic Ovary Syndrome. Biomed. Pharmacother..

[164] Lee H.C. (1997). Mechanisms of Calcium Signaling by Cyclic ADP-Ribose and NAADP. Physiol. Rev..

[165] Lee H.C. (2001). Physiological Functions of Cyclic ADP-Ribose and NAADP as Calcium Messengers. Annu. Rev. Pharmacol. Toxicol..

[166] Menteyne A., Burdakov A., Charpentier G., Petersen O.H., Cancela J.-M. (2006). Generation of Specific Ca(2+) Signals from Ca(2+) Stores and Endocytosis by Differential Coupling to Messengers. Curr. Biol..

[167] Chini C.C.S., Peclat T.R., Warner G.M., Kashyap S., Espindola-Netto J.M., de Oliveira G.C., Gomez L.S., Hogan K.A., Tarragó M.G., Puranik A.S. (2020). CD38 Ecto-Enzyme in Immune Cells Is Induced during Aging Regulating NAD^+^ and NMN Levels. Nat. Metab..

[168] Barbosa M.T.P., Soares S.M., Novak C.M., Sinclair D., Levine J.A., Aksoy P., Chini E.N. (2007). The Enzyme CD38 (a NAD Glycohydrolase, EC 3.2.2.5) Is Necessary for the Development of Diet-Induced Obesity. FASEB J..

[169] Morandi F., Airoldi I., Marimpietri D., Bracci C., Faini A.C., Gramignoli R. (2019). CD38, a Receptor with Multifunctional Activities: From Modulatory Functions on Regulatory Cell Subsets and Extracellular Vesicles, to a Target for Therapeutic Strategies. Cells.

[170] Covarrubias A.J., Kale A., Perrone R., Lopez-Dominguez J.A., Pisco A.O., Kasler H.G., Schmidt M.S., Heckenbach I., Kwok R., Wiley C.D. (2020). Senescent Cells Promote Tissue NAD^+^ Decline during Ageing via the Activation of CD38+ Macrophages. Nat. Metab..

[171] Deaglio S., Mallone R., Baj G., Donati D., Giraudo G., Corno F., Bruzzone S., Geuna M., Ausiello C., Malavasi F. (2001). Human CD38 and Its Ligand CD31 Define a Unique Lamina Propria T Lymphocyte Signaling Pathway. FASEB J..

[172] van de Donk N.W.C.J., Janmaat M.L., Mutis T., Lammerts van Bueren J.J., Ahmadi T., Sasser A.K., Lokhorst H.M., Parren P.W.H.I. (2016). Monoclonal Antibodies Targeting CD38 in Hematological Malignancies and Beyond. Immunol. Rev..

[173] Fang E.F., Kassahun H., Croteau D.L., Scheibye-Knudsen M., Marosi K., Lu H., Shamanna R.A., Kalyanasundaram S., Bollineni R.C., Wilson M.A. (2016). NAD^+^ Replenishment Improves Lifespan and Healthspan in Ataxia Telangiectasia Models via Mitophagy and DNA Repair. Cell Metab..

[174] Bai P., Canto C., Brunyánszki A., Huber A., Szántó M., Cen Y., Yamamoto H., Houten S.M., Kiss B., Oudart H. (2011). PARP-2 Regulates SIRT1 Expression and Whole-Body Energy Expenditure. Cell Metab..

[175] Pirinen E., Canto C., Jo Y.-S., Morato L., Zhang H., Menzies K., Williams E.G., Mouchiroud L., Moullan N., Hagberg C. (2014). Pharmacological Inhibition of Poly(ADP-Ribose) Polymerases Improves Fitness and Mitochondrial Function in Skeletal Muscle. Cell Metab..

[176] Scheibye-Knudsen M., Mitchell S.J., Fang E.F., Iyama T., Ward T., Wang J., Dunn C.A., Singh N., Veith S., Hasan-Olive M.M. (2014). A High-Fat Diet and NAD^+^ Activate Sirt1 to Rescue Premature Aging in Cockayne Syndrome. Cell Metab..

[177] Fang E.F., Scheibye-Knudsen M., Brace L.E., Kassahun H., SenGupta T., Nilsen H., Mitchell J.R., Croteau D.L., Bohr V.A. (2014). Defective Mitophagy in XPA via PARP1 Hyperactivation and NAD^+^/SIRT1 Reduction. Cell.

[178] Oliver F.J., Ménissier-de Murcia J., Nacci C., Decker P., Andriantsitohaina R., Muller S., de la Rubia G., Stoclet J.C., de Murcia G. (1999). Resistance to Endotoxic Shock as a Consequence of Defective NF-KappaB Activation in Poly (ADP-Ribose) Polymerase-1 Deficient Mice. EMBO J..

[179] Boughton-Smith N.K., Evans S.M., Hawkey C.J., Cole A.T., Balsitis M., Whittle B.J., Moncada S. (1993). Nitric Oxide Synthase Activity in Ulcerative Colitis and Crohn’s Disease. Lancet.

[180] Singer I.I., Kawka D.W., Scott S., Weidner J.R., Mumford R.A., Riehl T.E., Stenson W.F. (1996). Expression of Inducible Nitric Oxide Synthase and Nitrotyrosine in Colonic Epithelium in Inflammatory Bowel Disease. Gastroenterology.

[181] Brunyanszki A., Olah G., Coletta C., Szczesny B., Szabo C. (2014). Regulation of Mitochondrial Poly(ADP-Ribose) Polymerase Activation by the β-Adrenoceptor/CAMP/Protein Kinase A Axis during Oxidative Stress. Mol. Pharmacol..

[182] Lasry A., Zinger A., Ben-Neriah Y. (2016). Inflammatory Networks Underlying Colorectal Cancer. Nat. Immunol..

[183] Dörsam B., Seiwert N., Foersch S., Stroh S., Nagel G., Begaliew D., Diehl E., Kraus A., McKeague M., Minneker V. (2018). PARP-1 Protects against Colorectal Tumor Induction, but Promotes Inflammation-Driven Colorectal Tumor Progression. Proc. Natl. Acad. Sci. USA.

[184] Giannone P.J., Alcamo A.A., Schanbacher B.L., Nankervis C.A., Besner G.E., Bauer J.A. (2011). Poly(ADP-Ribose) Polymerase-1: A Novel Therapeutic Target in Necrotizing Enterocolitis. Pediatr. Res..

[185] Moschen A.R., Kaser A., Enrich B., Mosheimer B., Theurl M., Niederegger H., Tilg H. (2007). Visfatin, an Adipocytokine with Proinflammatory and Immunomodulating Properties. J. Immunol..

[186] Samal B., Sun Y., Stearns G., Xie C., Suggs S., McNiece I. (1994). Cloning and Characterization of the CDNA Encoding a Novel Human Pre-B-Cell Colony-Enhancing Factor. Mol. Cell. Biol..

[187] Li Y., Zhang Y., Dorweiler B., Cui D., Wang T., Woo C.W., Brunkan C.S., Wolberger C., Imai S., Tabas I. (2008). Extracellular Nampt Promotes Macrophage Survival via a Nonenzymatic Interleukin-6/STAT3 Signaling Mechanism. J. Biol. Chem..

[188] Van den Bergh R., Morin S., Sass H.J., Grzesiek S., Vekemans M., Florence E., Thanh Thi Tran H., Imiru R.G., Heyndrickx L., Vanham G. (2012). Monocytes Contribute to Differential Immune Pressure on R5 versus X4 HIV through the Adipocytokine Visfatin/NAMPT. PLoS ONE.

[189] Camp S.M., Ceco E., Evenoski C.L., Danilov S.M., Zhou T., Chiang E.T., Moreno-Vinasco L., Mapes B., Zhao J., Gursoy G. (2015). Unique Toll-Like Receptor 4 Activation by NAMPT/PBEF Induces NFκB Signaling and Inflammatory Lung Injury. Sci. Rep..

[190] Managò A., Audrito V., Mazzola F., Sorci L., Gaudino F., Gizzi K., Vitale N., Incarnato D., Minazzato G., Ianniello A. (2019). Extracellular Nicotinate Phosphoribosyltransferase Binds Toll like Receptor 4 and Mediates Inflammation. Nat. Commun..

[191] Colombo G., Travelli C., Porta C., Genazzani A.A. (2022). Extracellular Nicotinamide Phosphoribosyltransferase Boosts IFNγ-Induced Macrophage Polarization Independently of TLR4. Science.

[192] Grolla A.A., Travelli C., Genazzani A.A., Sethi J.K. (2016). Extracellular Nicotinamide Phosphoribosyltransferase, a New Cancer Metabokine. Br. J. Pharmacol..

[193] Galassi L., Di Stefano M., Brunetti L., Orsomando G., Amici A., Ruggieri S., Magni G. (2012). Characterization of Human Nicotinate Phosphoribosyltransferase: Kinetic Studies, Structure Prediction and Functional Analysis by Site-Directed Mutagenesis. Biochimie.

[194] Collins P.B., Chaykin S. (1972). The Management of Nicotinamide and Nicotinic Acid in the Mouse. J. Biol. Chem..

[195] Hara N., Yamada K., Shibata T., Osago H., Tsuchiya M. (2011). Nicotinamide Phosphoribosyltransferase/Visfatin Does Not Catalyze Nicotinamide Mononucleotide Formation in Blood Plasma. PLoS ONE.

[196] Gaut Z.N., Solomon H.M. (1971). Inhibition of Nicotinate Phosphoribosyltransferase in Human Platelet Lysate by Nicotinic Acid Analogs. Biochem. Pharmacol..

[197] Ruggieri S., Orsomando G., Sorci L., Raffaelli N. (2015). Regulation of NAD Biosynthetic Enzymes Modulates NAD-Sensing Processes to Shape Mammalian Cell Physiology under Varying Biological Cues. Biochim. Biophys. Acta.

[198] Smith L.D., Gholson R.K. (1969). Allosteric Properties of Bovine Liver Nicotinate Phosphoribosyltransferase. J. Biol. Chem..

[199] Neubauer K., Bednarz-Misa I., Walecka-Zacharska E., Wierzbicki J., Agrawal A., Gamian A., Krzystek-Korpacka M. (2019). Oversecretion and Overexpression of Nicotinamide Phosphoribosyltransferase/Pre-B Colony-Enhancing Factor/Visfatin in Inflammatory Bowel Disease Reflects the Disease Activity, Severity of Inflammatory Response and Hypoxia. Int. J. Mol. Sci..

[200] Colombo G., Caviglia G.P., Ravera A., Tribocco E., Frara S., Rosso C., Travelli C., Genazzani A.A., Ribaldone D.G. (2023). NAMPT and NAPRT Serum Levels Predict Response to Anti-TNF Therapy in Inflammatory Bowel Disease. Front. Med. (Lausanne).

[201] Piacente F., Caffa I., Ravera S., Sociali G., Passalacqua M., Vellone V.G., Becherini P., Reverberi D., Monacelli F., Ballestrero A. (2017). Nicotinic Acid Phosphoribosyltransferase Regulates Cancer Cell Metabolism, Susceptibility to NAMPT Inhibitors, and DNA Repair. Cancer Res..

[202] Roberti A., Fernández A.F., Fraga M.F. (2021). Nicotinamide N-Methyltransferase: At the Crossroads between Cellular Metabolism and Epigenetic Regulation. Mol. Metab..

[203] Peng Y., Sartini D., Pozzi V., Wilk D., Emanuelli M., Yee V.C. (2011). Structural Basis of Substrate Recognition in Human Nicotinamide N-Methyltransferase. Biochemistry.

[204] Aksoy S., Szumlanski C.L., Weinshilboum R.M. (1994). Human Liver Nicotinamide N-Methyltransferase. CDNA Cloning, Expression, and Biochemical Characterization. J. Biol. Chem..

[205] Smith M.L., Burnett D., Bennett P., Waring R.H., Brown H.M., Williams A.C., Ramsden D.B. (1998). A Direct Correlation between Nicotinamide N-Methyltransferase Activity and Protein Levels in Human Liver Cytosol. Biochim. Biophys. Acta.

[206] Seifert R., Hoshino J., Kröger H. (1984). Nicotinamide Methylation. Tissue Distribution, Developmental and Neoplastic Changes. Biochim. Biophys. Acta.

[207] Campagna R., Mateuszuk Ł., Wojnar-Lason K., Kaczara P., Tworzydło A., Kij A., Bujok R., Mlynarski J., Wang Y., Sartini D. (2021). Nicotinamide N-Methyltransferase in Endothelium Protects against Oxidant Stress-Induced Endothelial Injury. Biochim. Biophys. Acta Mol. Cell Res..

[208] Riederer M., Erwa W., Zimmermann R., Frank S., Zechner R. (2009). Adipose Tissue as a Source of Nicotinamide N-Methyltransferase and Homocysteine. Atherosclerosis.

[209] Xu J., Capezzone M., Xu X., Hershman J.M. (2005). Activation of Nicotinamide N-Methyltransferase Gene Promoter by Hepatocyte Nuclear Factor-1beta in Human Papillary Thyroid Cancer Cells. Mol. Endocrinol..

[210] Katsyuba E., Auwerx J. (2017). Modulating NAD + Metabolism, from Bench to Bedside. EMBO J..

[211] Xie X., Yu H., Wang Y., Zhou Y., Li G., Ruan Z., Li F., Wang X., Liu H., Zhang J. (2014). Nicotinamide N-Methyltransferase Enhances the Capacity of Tumorigenesis Associated with the Promotion of Cell Cycle Progression in Human Colorectal Cancer Cells. Arch. Biochem. Biophys..

[212] Lu X.M., Long H. (2018). Nicotinamide N-Methyltransferase as a Potential Marker for Cancer. Neoplasma.

[213] Ogawa M., Tanaka A., Namba K., Shia J., Wang J.Y., Roehrl M.H.A. (2022). Tumor Stromal Nicotinamide N-Methyltransferase Overexpression as a Prognostic Biomarker for Poor Clinical Outcome in Early-Stage Colorectal Cancer. Sci. Rep..

[214] Neelakantan H., Brightwell C.R., Graber T.G., Maroto R., Leo Wang H.-Y., McHardy S.F., Papaconstantinou J., Fry C.S., Watowich S.J. (2019). Small Molecule Nicotinamide N-Methyltransferase Inhibitor Activates Senescent Muscle Stem Cells and Improves Regenerative Capacity of Aged Skeletal Muscle. Biochem. Pharmacol..

[215] Liu A., Guo M., He L., Martínez M.-A., Martínez M., Lopez-Torres B., Martínez-Larrañaga M.-R., Wang X., Anadón A., Ares I. (2022). Nicotinamide N-Methyltransferase Protects against Deoxynivalenol-Induced Growth Inhibition by Suppressing pro-Inflammatory Cytokine Expression. Food Chem. Toxicol..

[216] Jakubowski A., Sternak M., Jablonski K., Ciszek-Lenda M., Marcinkiewicz J., Chlopicki S. (2016). 1-Methylnicotinamide Protects against Liver Injury Induced by Concanavalin A via a Prostacyclin-Dependent Mechanism: A Possible Involvement of IL-4 and TNF-α. Int. Immunopharmacol..

[217] Komatsu M., Kanda T., Urai H., Kurokochi A., Kitahama R., Shigaki S., Ono T., Yukioka H., Hasegawa K., Tokuyama H. (2018). NNMT Activation Can Contribute to the Development of Fatty Liver Disease by Modulating the NAD^+^ Metabolism. Sci. Rep..

[218] Andrieux P., Chevillard C., Cunha-Neto E., Nunes J.P.S. (2021). Mitochondria as a Cellular Hub in Infection and Inflammation. Int. J. Mol. Sci..

[219] Vragović J., Vraţić H. (2016). Inflammatory Bowel Disease. Prog. Drug Res..

[220] Fritze C.E., Verschueren K., Strich R., Easton Esposito R. (1997). Direct Evidence for SIR2 Modulation of Chromatin Structure in Yeast RDNA. EMBO J..

[221] Bryan S., Baregzay B., Spicer D., Singal P.K., Khaper N. (2013). Redox-Inflammatory Synergy in the Metabolic Syndrome. Can. J. Physiol. Pharmacol..

[222] Lautrup S., Sinclair D.A., Mattson M.P., Fang E.F. (2019). NAD^+^ in Brain Aging and Neurodegenerative Disorders. Cell Metab..

[223] Baixauli F., Acín-Pérez R., Villarroya-Beltrí C., Mazzeo C., Nuñez-Andrade N., Gabandé-Rodriguez E., Dolores Ledesma M., Blázquez A., Martin M.A., Falcón-Pérez J.M. (2015). Mitochondrial Respiration Controls Lysosomal Function during Inflammatory T Cell Responses. Cell Metab..

[224] Gomes A.P., Price N.L., Ling A.J.Y., Moslehi J.J., Montgomery M.K., Rajman L., White J.P., Teodoro J.S., Wrann C.D., Hubbard B.P. (2013). Declining NAD^+^ Induces a Pseudohypoxic State Disrupting Nuclear-Mitochondrial Communication during Aging. Cell.

[225] Minhas P.S., Liu L., Moon P.K., Joshi A.U., Dove C., Mhatre S., Contrepois K., Wang Q., Lee B.A., Coronado M. (2019). Macrophage de Novo NAD^+^ Synthesis Specifies Immune Function in Aging and Inflammation. Nat. Immunol..

[226] Mouchiroud L., Houtkooper R.H., Moullan N., Katsyuba E., Ryu D., Cantó C., Mottis A., Jo Y.-S., Viswanathan M., Schoonjans K. (2013). The NAD^+^/Sirtuin Pathway Modulates Longevity through Activation of Mitochondrial UPR and FOXO Signaling. Cell.

[227] Karamanlidis G., Lee C.F., Garcia-Menendez L., Kolwicz S.C., Suthammarak W., Gong G., Sedensky M.M., Morgan P.G., Wang W., Tian R. (2013). Mitochondrial Complex I Deficiency Increases Protein Acetylation and Accelerates Heart Failure. Cell Metab..

[228] Desdín-Micó G., Soto-Heredero G., Aranda J.F., Oller J., Carrasco E., Gabandé-Rodríguez E., Blanco E.M., Alfranca A., Cussó L., Desco M. (2020). T Cells with Dysfunctional Mitochondria Induce Multimorbidity and Premature Senescence. Science.

[229] Almeida L., Dhillon-LaBrooy A., Castro C.N., Adossa N., Carriche G.M., Guderian M., Lippens S., Dennerlein S., Hesse C., Lambrecht B.N. (2021). Ribosome-Targeting Antibiotics Impair T Cell Effector Function and Ameliorate Autoimmunity by Blocking Mitochondrial Protein Synthesis. Immunity.

[230] Roediger W.E. (1980). The Colonic Epithelium in Ulcerative Colitis: An Energy-Deficiency Disease?. Lancet.

[231] Haberman Y., Karns R., Dexheimer P.J., Schirmer M., Somekh J., Jurickova I., Braun T., Novak E., Bauman L., Collins M.H. (2019). Ulcerative Colitis Mucosal Transcriptomes Reveal Mitochondriopathy and Personalized Mechanisms Underlying Disease Severity and Treatment Response. Nat. Commun..

[232] Smith S.A., Ogawa S.A., Chau L., Whelan K.A., Hamilton K.E., Chen J., Tan L., Chen E.Z., Keilbaugh S., Fogt F. (2020). Mitochondrial Dysfunction in Inflammatory Bowel Disease Alters Intestinal Epithelial Metabolism of Hepatic Acylcarnitines. J. Clin. Investig..

[233] Graham D.B., Xavier R.J. (2020). Pathway Paradigms Revealed from the Genetics of Inflammatory Bowel Disease. Nature.

[234] Jostins L., Ripke S., Weersma R.K., Duerr R.H., McGovern D.P., Hui K.Y., Lee J.C., Schumm L.P., Sharma Y., Anderson C.A. (2012). Host-Microbe Interactions Have Shaped the Genetic Architecture of Inflammatory Bowel Disease. Nature.

[235] Lahiri A., Hedl M., Yan J., Abraham C. (2017). Human LACC1 Increases Innate Receptor-Induced Responses and a LACC1 Disease-Risk Variant Modulates These Outcomes. Nat. Commun..

[236] Muise A.M., Xu W., Guo C.-H., Walters T.D., Wolters V.M., Fattouh R., Lam G.Y., Hu P., Murchie R., Sherlock M. (2012). NADPH Oxidase Complex and IBD Candidate Gene Studies: Identification of a Rare Variant in NCF2 That Results in Reduced Binding to RAC2. Gut.

[237] Rivas M.A., Beaudoin M., Gardet A., Stevens C., Sharma Y., Zhang C.K., Boucher G., Ripke S., Ellinghaus D., Burtt N. (2011). Deep Resequencing of GWAS Loci Identifies Independent Rare Variants Associated with Inflammatory Bowel Disease. Nat. Genet..

[238] Suzuki T. (2013). Regulation of Intestinal Epithelial Permeability by Tight Junctions. Cell. Mol. Life Sci..

[239] Garcia-Hernandez V., Quiros M., Nusrat A. (2017). Intestinal Epithelial Claudins: Expression and Regulation in Homeostasis and Inflammation. Ann. N. Y. Acad. Sci..

[240] Tsukita S., Furuse M., Itoh M. (2001). Multifunctional Strands in Tight Junctions. Nat. Rev. Mol. Cell Biol..

[241] Anderson J.M., Van Itallie C.M. (1995). Tight Junctions and the Molecular Basis for Regulation of Paracellular Permeability. Am. J. Physiol..

[242] Unno N., Fink M.P. (1998). Intestinal Epithelial Hyperpermeability. Mechanisms and Relevance to Disease. Gastroenterol. Clin. N. Am..

[243] Khan A.U., Delude R.L., Han Y.Y., Sappington P.L., Han X., Carcillo J.A., Fink M.P. (2002). Liposomal NAD(^+^) Prevents Diminished O(2) Consumption by Immunostimulated Caco-2 Cells. Am. J. Physiol. Lung Cell. Mol. Physiol..

[244] Bai M., Lu C., An L., Gao Q., Xie W., Miao F., Chen X., Pan Y., Wang Q. (2020). SIRT1 Relieves Necrotizing Enterocolitis through Inactivation of Hypoxia-Inducible Factor (HIF)-1a. Cell Cycle.

[245] Berger F., Ramírez-Hernández M.H., Ziegler M. (2004). The New Life of a Centenarian: Signalling Functions of NAD(P). Trends Biochem. Sci..

[246] Pollak N., Dölle C., Ziegler M. (2007). The Power to Reduce: Pyridine Nucleotides–Small Molecules with a Multitude of Functions. Biochem. J..

[247] Jaiswal A.K. (2000). Regulation of Genes Encoding NAD(P)H:Quinone Oxidoreductases. Free Radic. Biol. Med..

[248] Nam S.T., Hwang J.H., Kim D.H., Park M.J., Lee I.H., Nam H.J., Kang J.K., Kim S.K., Hwang J.S., Chung H.K. (2014). Role of NADH: Quinone Oxidoreductase-1 in the Tight Junctions of Colonic Epithelial Cells. BMB Rep..

[249] Folmes C.D.L., Dzeja P.P., Nelson T.J., Terzic A. (2012). Metabolic Plasticity in Stem Cell Homeostasis and Differentiation. Cell Stem Cell.

[250] Zhang H., Menzies K.J., Auwerx J. (2018). The Role of Mitochondria in Stem Cell Fate and Aging. Development.

[251] Brown K., Xie S., Qiu X., Mohrin M., Shin J., Liu Y., Zhang D., Scadden D.T., Chen D. (2013). SIRT3 Reverses Aging-Associated Degeneration. Cell Rep..

[252] Mohrin M., Shin J., Liu Y., Brown K., Luo H., Xi Y., Haynes C.M., Chen D. (2015). STEM CELL AGING. A Mitochondrial UPR-Mediated Metabolic Checkpoint Regulates Hematopoietic Stem Cell Aging. Science.

[253] Zhang H., Ryu D., Wu Y., Gariani K., Wang X., Luan P., D’Amico D., Ropelle E.R., Lutolf M.P., Aebersold R. (2016). NAD^+^ Repletion Improves Mitochondrial and Stem Cell Function and Enhances Life Span in Mice. Science.

[254] Biteau B., Hochmuth C.E., Jasper H. (2011). Maintaining Tissue Homeostasis: Dynamic Control of Somatic Stem Cell Activity. Cell Stem Cell.

[255] Barker N., Tan S., Clevers H. (2013). Lgr Proteins in Epithelial Stem Cell Biology. Development.

[256] Barker N., van Es J.H., Kuipers J., Kujala P., van den Born M., Cozijnsen M., Haegebarth A., Korving J., Begthel H., Peters P.J. (2007). Identification of Stem Cells in Small Intestine and Colon by Marker Gene Lgr5. Nature.

[257] Mihaylova M.M., Cheng C.-W., Cao A.Q., Tripathi S., Mana M.D., Bauer-Rowe K.E., Abu-Remaileh M., Clavain L., Erdemir A., Lewis C.A. (2018). Fasting Activates Fatty Acid Oxidation to Enhance Intestinal Stem Cell Function during Homeostasis and Aging. Cell Stem Cell.

[258] Nalapareddy K., Nattamai K.J., Kumar R.S., Karns R., Wikenheiser-Brokamp K.A., Sampson L.L., Mahe M.M., Sundaram N., Yacyshyn M.-B., Yacyshyn B. (2017). Canonical Wnt Signaling Ameliorates Aging of Intestinal Stem Cells. Cell Rep..

[259] Annunziata F., Rasa S.M.M., Krepelova A., Lu J., Minetti A., Omrani O., Nunna S., Adam L., Käppel S., Neri F. (2022). Paneth Cells Drive Intestinal Stem Cell Competition and Clonality in Aging and Calorie Restriction. Eur. J. Cell Biol..

[260] Navas L.E., Carnero A. (2021). NAD^+^ Metabolism, Stemness, the Immune Response, and Cancer. Signal. Transduct. Target Ther..

[261] Hong S.M., Lee A.-Y., Hwang S.-M., Ha Y.-J., Kim M.-J., Min S., Hwang W., Yoon G., Kwon S.M., Woo H.G. (2022). NAMPT Mitigates Colitis Severity by Supporting Redox-Sensitive Activation of Phagocytosis in Inflammatory Macrophages. Redox Biol..

[262] Peritore A.F., D’Amico R., Cordaro M., Siracusa R., Fusco R., Gugliandolo E., Genovese T., Crupi R., Di Paola R., Cuzzocrea S. (2021). PEA/Polydatin: Anti-Inflammatory and Antioxidant Approach to Counteract DNBS-Induced Colitis. Antioxidants.

[263] Xiong Y., Shi L., Wang L., Zhou Z., Wang C., Lin Y., Luo D., Qiu J., Chen D. (2017). Activation of Sirtuin 1 by Catalpol-Induced down-Regulation of MicroRNA-132 Attenuates Endoplasmic Reticulum Stress in Colitis. Pharmacol. Res..

[264] Akimova T., Xiao H., Liu Y., Bhatti T.R., Jiao J., Eruslanov E., Singhal S., Wang L., Han R., Zacharia K. (2014). Targeting Sirtuin-1 Alleviates Experimental Autoimmune Colitis by Induction of Foxp3+ T-Regulatory Cells. Mucosal. Immunol..

[265] Mabley J.G., Jagtap P., Perretti M., Getting S.J., Salzman A.L., Virág L., Szabó E., Soriano F.G., Liaudet L., Abdelkarim G.E. (2001). Anti-Inflammatory Effects of a Novel, Potent Inhibitor of Poly (ADP-Ribose) Polymerase. Inflamm. Res..

[266] Zingarelli B., O’Connor M., Hake P.W. (2003). Inhibitors of Poly (ADP-Ribose) Polymerase Modulate Signal Transduction Pathways in Colitis. Eur. J. Pharmacol..

[267] Sánchez-Fidalgo S., Villegas I., Martín A., Sánchez-Hidalgo M., Alarcón de la Lastra C. (2007). PARP Inhibition Reduces Acute Colonic Inflammation in Rats. Eur. J. Pharmacol..

[268] Lu H., Lin J., Xu C., Sun M., Zuo K., Zhang X., Li M., Huang H., Li Z., Wu W. (2021). Cyclosporine Modulates Neutrophil Functions via the SIRT6-HIF-1α-Glycolysis Axis to Alleviate Severe Ulcerative Colitis. Clin. Transl. Med..

[269] Huang P., Wang X., Wang S., Wu Z., Zhou Z., Shao G., Ren C., Kuang M., Zhou Y., Jiang A. (2022). Treatment of Inflammatory Bowel Disease: Potential Effect of NMN on Intestinal Barrier and Gut Microbiota. Curr. Res. Food Sci..

[270] Malhi G., Rumman A., Thanabalan R., Croitoru K., Silverberg M.S., Hillary Steinhart A., Nguyen G.C. (2015). Vaccination in Inflammatory Bowel Disease Patients: Attitudes, Knowledge, and Uptake. J. Crohn’s Colitis.

[271] Murray M.F., Nghiem M., Srinivasan A. (1995). HIV Infection Decreases Intracellular Nicotinamide Adenine Dinucleotide [NAD]. Biochem. Biophys. Res. Commun..

[272] Vanham G., Toossi Z., Hirsch C.S., Wallis R.S., Schwander S.K., Rich E.A., Ellner J.J. (1997). Examining a Paradox in the Pathogenesis of Human Pulmonary Tuberculosis: Immune Activation and Suppression/Anergy. Tuber. Lung Dis..

[273] Rozwarski D.A., Grant G.A., Barton D.H., Jacobs W.R., Sacchettini J.C. (1998). Modification of the NADH of the Isoniazid Target (InhA) from Mycobacterium Tuberculosis. Science.

